# Strong Small‐Scale Differentiation but No Cryptic Species Within the Two Isopod Species *Asellus aquaticus* and *Proasellus coxalis* in a Restored Urban River System (Emscher, Germany)

**DOI:** 10.1002/ece3.70575

**Published:** 2024-11-18

**Authors:** Martina Weiss, Armin W. Lorenz, Christian K. Feld, Florian Leese

**Affiliations:** ^1^ Aquatic Ecosystem Research University of Duisburg‐Essen Essen Germany; ^2^ Centre for Water and Environmental Research (ZWU) University of Duisburg‐Essen Essen Germany; ^3^ Aquatic Ecology University of Duisburg‐Essen Essen Germany

**Keywords:** intraspecific genetic diversity, metapopulation, population genetics, recolonization, species delimitation, stream restoration

## Abstract

Worldwide, humans have strongly altered river networks. Key changes resulted in modified hydromorphology, poor habitat quality and availability, migration barriers, and pollution. Restoration measures aim at mitigating anthropogenic stressors and at restoring connectivity, but the biological success of these measures is not guaranteed. Analyzing genetic diversity and metapopulation structure of target species in the river network with genetic markers can help to understand recolonization processes and to identify persisting gene flow barriers. Here, we studied the population genetic structure of the two pollution‐tolerant detritivorous isopod species, *Asellus aquaticus* and *Proasellus coxalis* , in the former heavily degraded and polluted, but now mostly restored Emscher catchment in Germany. For both species, we analyzed mitochondrial cytochrome c oxidase I (COI) gene sequences and nuclear genome‐wide single nucleotide polymorphism (SNP) data. Surprisingly, we found a strong metapopulation structure for both species with several isolated populations on a small‐scale of few kilometers, but a still high genetic diversity, especially in the COI gene. For both taxa, potentially cryptic species are known, but our SNP data showed that the mitochondrial lineages represent only one species, each, in the study area. This highlights the importance of integrating high‐resolution nuclear markers into species identification because species diversity may otherwise be overestimated. While we could identify some migration barriers and find indications for passive dispersal by birds or humans, these factors could not fully explain the local metapopulation structure, suggesting that also other drivers, such as isolation by adaptation, priority effects, or biotic interactions, play a role in shaping the population genetic structure.

## Graphical abstract

We studied the metapopulation structure of two strictly aquatic isopod species in a restored urban stream catchment using mitochondrial COI and genome‐wide SNP data. Both species showed a substantial small‐scale metapopulation structure, which could be partly explained by migration barriers and passive dispersal by birds or humans.

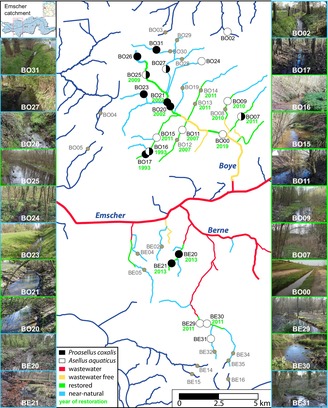

## 1 Introduction

As the lowest‐lying areas of the landscape, rivers and streams integrate the effects of land‐use change and are therefore especially sensitive to and strongly impacted by the changes associated with the urbanization of catchments (Bernhardt and Palmer 2007). To fit in the urban structure, the original riparian vegetation has often been replaced by impervious surfaces such as asphalt or concrete, thus increasing the surface run‐off and substance load entering urban streams (Bernhardt and Palmer 2007; Gillmann, Hering, and Lorenz 2023). For centuries, urban streams have been regulated and modified into mostly straight channels to increase discharge (flood prevention) and to limit the area they occupy. Consequently, urban stream hydromorphology has changed widespread and substantially. The increasing awareness of aquatic biodiversity and its value in concert with a wide range of ecosystem services provided by near‐natural rivers and floodplains has resulted in numerous river restoration projects (Jähnig et al. 2011), including urban streams (Gillmann, Hering, and Lorenz 2023; Winking et al. 2014). However, effects of river restoration on benthic invertebrates, which are frequently used to assess the ecological status of rivers and streams, are often minor (e.g., Jähnig et al. 2010; Lorenz and Feld 2013; Pilotto et al. 2019; Sundermann, Stoll, and Haase 2011). Sundermann, Stoll, and Haase (2011) found that the initial (successful) recolonization depends on the species pool that is available in the near surroundings (< 5 km) of restored sites. But also the species‐specific dispersal capacity may constitute a major determinant of the successful recolonization of restored stream sections (Gillmann et al. 2024; Hughes 2007; Jähnig et al. 2010; Sundermann, Stoll, and Haase 2011; Winking et al. 2014). The dispersal capacity can be estimated from species life history and dispersal traits (e.g., merolimnic vs. hololimnic, active dispersal vs. passive drift; Li, Tonkin, and Haase 2018; Sarremejane et al. 2020) and empirical evidence on realized dispersal distances (e.g., Sondermann et al. 2017; Winking et al. 2014). As a source for recolonization of hololimnic, that is, fully aquatic species, immigration by drift from connected upstream sites is often assumed to be the major pathway (e.g., Hughes 2007; Winking et al. 2014). However, while estimated dispersal capacity and time needed to recolonize a restored stream section may correlate, connectivity or gene flow between populations is still difficult to predict (e.g., Weiss, Weigand, and Leese 2022; Weiss and Leese 2016). Besides area‐ and site‐specific factors, such as water pollution, in‐stream and terrestrial migration barriers, or anthropogenic land use, intra‐ and interspecific competition and evolutionary adaptation can play a role in shaping realized gene flow (e.g., de Meester et al. 2002; Fraser, Banks, and Waters 2014). However, connectivity between populations after initial recolonization is important to maintain genetic diversity because, in small, isolated populations, genetic diversity is lost due to genetic drift. The loss of genetic diversity can reduce the capacity of populations to adapt to changing environments (Bijlsma and Loeschcke 2012; Frankham 2010, 2005; Hughes et al. 2008; Reusch et al. 2005). To better understand the recolonization process and to evaluate its success, it is important to analyze the connectivity among populations and to recognize migration barriers. These aims can be achieved by analyzing high‐density genomic markers, such as single nucleotide polymorphisms (SNPs) distributed across the whole genome (e.g., Fuller et al. 2019; Miles et al. 2019). Based on such genetic markers it is possible to study population structure of aquatic invertebrates at local and regional scales due to the impacts of genetic drift in isolation or after a founding event (e.g., Angst et al. 2022; Montero‐Pau, Gómez, and Serra 2018; Weiss, Weigand, and Leese 2022). Different genotyping‐by‐sequencing approaches exist to generate such SNP data, one popular and powerful being double‐digest restriction site‐associated DNA sequencing (ddRAD‐seq, Peterson et al. 2012).

One example of a former strongly degraded and polluted river system is the Emscher catchment located in the “Ruhr Metropolitan Area” (Western Germany), one of the densest urban agglomerations in Europe (Gerner et al. 2018). In a 30‐year‐long project starting in 1990, the Emscher and in particular its tributaries have been restored from a highly modified open wastewater channel system with concrete beds into a wastewater‐free, partly near‐natural stream system with sinuating or semi‐meandering river courses and naturally developed riparian vegetation (Gerner et al. 2018; Gillmann, Hering, and Lorenz 2023). One key species group, important for ecosystem functioning like organic matter decomposition and therefore important to return after restoration, are detritivorous species such as amphipods and isopods. One isopod species that can quickly recolonize restored stream sections or even persist in polluted water is *Asellus aquaticus* (Gillmann, Hering, and Lorenz 2023). *A*. *aquaticus* is opportunistic in waters with various physiochemical conditions, has been found to be relatively pollution tolerant to organic and chemical pollution, and resilient to low oxygen levels and fairly high concentrations of heavy metals in various studies (e.g., Basset 1993; Fraser, Parkin, and Verspoor 1978; Hervant and Malard 2019; MacNeil et al. 2002; Maltby 1995; van Ginneken, Blust, and Bervoets 2019, 2017). Furthermore, *A*. *aquaticus* has been found to be a diverse species complex, comprising several potential cryptic species (Sworobowicz et al. 2020, 2015; Verovnik, Sket, and Trontelj 2005, 2004). Two of these (OTU A and J, sensu Sworobowicz et al. 2015) have been found with many haplotypes in the Emscher catchment in a study, in which the correlation of intraspecific genetic diversity of benthic invertebrates with stream degradation was assessed using a metabarcoding approach (Zizka, Weiss, and Leese 2020). The high genetic diversity, which has been found in the barcoding fragment of the mitochondrial cytochrome c oxidase I (COI) gene throughout Europe (e.g., Sworobowicz et al. 2020; Verovnik, Sket, and Trontelj 2005), was mainly explained by survival during glaciations not only in lower‐latitude refugia, which has been suggested for many terrestrial and freshwater species (e.g., Hewitt 1999; Taberlet et al. 1998), but also in numerous high‐latitude refugia (Sworobowicz et al. 2020). However, while many studies focusing on ecological or evolutionary aspects of *A*. *aquaticus* exist (reviewed in, e.g., Lafuente et al. 2021; O'Callaghan et al. 2019), little is known about small‐scale connectivity and realized gene flow between populations.

In addition to *A*. *aquaticus* , another isopod species, *Proasellus coxalis* , which is also relatively pollution tolerant (Spänhoff et al. 2007), occurs in the Emscher catchment. *P*. *coxalis* is thought to have originated from Southern Europe and has been first found in the river Rhine in Germany in 1931 (Bernauer and Jansen 2006), while *A*. *aquaticus* had recolonized northern Europe earlier after glaciation. Similar to *A*. *aquaticus* , it has been found to be a widely distributed, morphologically and genetically variable species complex with many described subspecies or molecular operational taxonomic units (MOTUs) (Eme et al. 2018; Ketmaier 2002; Morvan et al. 2013; Saclier et al. 2024; Stoch, Valentino, and Volpi 1996), which might be potential cryptic species. While different ecological aspects of the species have been studied (e.g., Basset and Rossi 1987; Cerfolli and Rossi 1995; Rossi, Basset, and Nobile 1983), less is known about realized gene flow on a small geographic scale. A study, analyzing 15 populations of the *P*. *coxalis* group in coastal and inland areas of Central Italy with allozyme data showed that on smaller geographic scales in the coastal areas, population were isolated by distance, while this pattern was less clear and a higher degree of genetic differentiation was found in the inland area (Ketmaier 2002). *A*. *aquaticus* and *P*. *coxalis* have similar ecological niches and are known to co‐occur with no consistent competitive advantage of one species over the other, but a rather complex relationship that can change according to locality and environmental conditions (Kemp et al. 2020).

In this study, we aimed to assess and compare the small‐scale population genomic structure of two freshwater isopod species with different recolonization histories, *A*. *aquaticus* and *P*. *coxalis* , in the restored urban river system of the Emscher catchment using two different genetic markers. While COI sequences were generated to identify the MOTUs of both species and to get an overview of the population structure, high‐resolution genomic SNPs, generated with ddRAD‐seq, were used to test if divergent MOTUs represent different cryptic species and to analyze connectivity and gene flow among populations. With this, our aim was to test the following hypotheses:

1.
*A*. *aquaticus* has a high historic (COI) and contemporary (ddRAD) genetic diversity and shows a high connectivity between stream sites within subcatchments in accordance with an isolation‐by‐distance (IBD) pattern.
2.
*P*. *coxalis* , which recolonized northern Europe more recently, shows a comparably low genetic diversity (especially in the COI gene) but also high levels of connectivity, with more gene flow between geographically close sites (IBD). This hypothesis is based on the fact that *P*. *coxalis* has not been present in the area as long as *A*. *aquaticus* , but is considered to be similarly pollution tolerant.

## 2 Materials and Methods

### 2.1 Sampling

**FIGURE 1:**
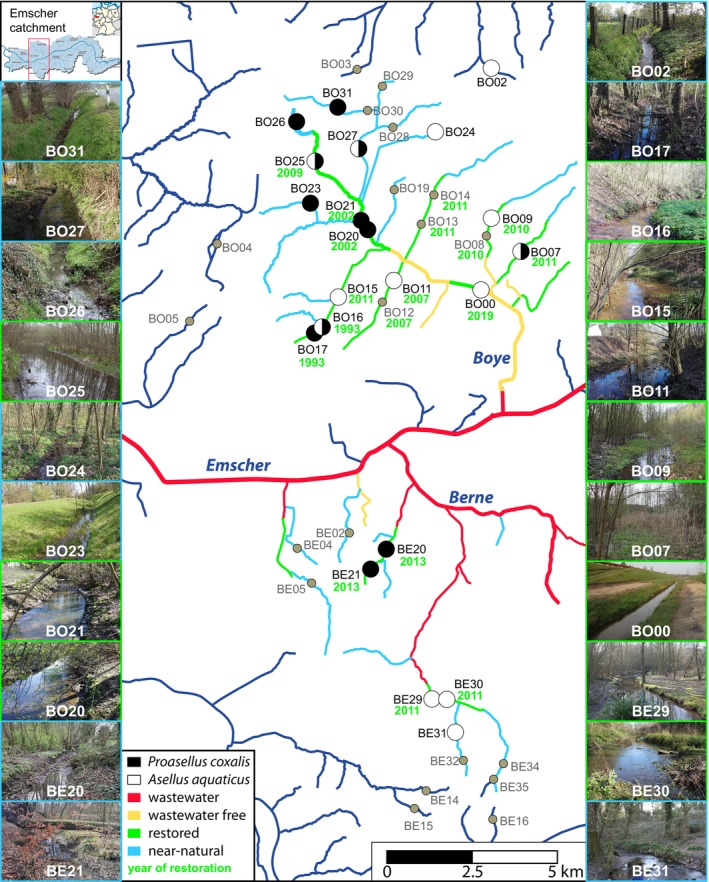
Location of sampling sites within Boye and Berne catchment with indication where *A*. *aquaticus* and *P*. *coxalis* were found. Furthermore, the ecological status in terms of restoration in 2019/2020 and the year of the restoration are shown below sampling sites, where the species were found. On the side, exemplary pictures (provided by Lea Heberle) and the location of the sampling area^*1^ in Germany^*2^ are shown. *1 (https://www.umwelt.nrw.de/system/files/media/images/2015‐07/Karte.jpg); *2 (https://en.m.wikipedia.org/wiki/File:Locator_map_RVR_in_Germany.svg).

Sampling sites were located in the Emscher catchment, which has a size of 775 km^2^. The Emscher is a right tributary to the river Rhine and has several larger tributaries, such as the Berne (catchment area 62 km^2^) and the Boye (catchment area 75 km^2^). Whereas most of the Boye catchment is already restored, parts of the Berne system still contain wastewater (Figure 1). The sampling sites were mainly located in tributaries north and south of the Emscher main stem with some additional sites located in adjacent catchments (Table S1). Most of the sites north of the Emscher belonged to the Boye catchment (23 of 27), while most sites south of the Emscher were located in the Berne catchment (8 of 14, see Table S1 for details). Regardless of the catchment, all sites north of the Emscher are abbreviated with BO, while the south sites are abbreviated with BE. The BO sites were mostly congruent with sites sampled in Winking et al. (2014). The sampling was conducted in March and April 2019 and all sites were revisited in March and April 2020. As mentioned in the introduction, both focal species of this study are known species complexes. As assignment to species within these complexes is only possible after the genetic analysis, we sampled *Asellus aquaticus* sensu lato and *Proasellus coxalis* sensu lato, but referred to them in the following as *A*. *aquaticus* and *P*. *coxalis* for better readability. In total, we visited 41 sites, but *A*. *aquaticus* and *P*. *coxalis* , were only found at 21 of these sites, *P*. *coxalis* only in Berne and Boye catchment, and *A*. *aquaticus* additionally at one site belonging to the Lippe catchment. While they were detected at four of these sites in sympatry, *A*. *aquaticus* was exclusively found at nine sites and *P*. *coxalis* at eight sites (Figure 1, Table S1). In 2019, we aimed at sampling 10 isopod specimens per site, while we increased sampling effort in 2020, aiming at 15 specimens per site. However, sampling success differed between years and sites in occurrences as well as in numbers, finally leading to 13 sites for *A*. *aquaticus* and 12 sites for *P*. *coxalis* (4 and 5 only 1 year, respectively; Table S1). In both years, the organisms were sampled using sieves and kick nets, preserved in 96% ethanol, and stored at 4°C until further processing.

### 2.2 Genotyping

Prior to DNA extraction, specimens were morphologically identified. To distinguish between *A*. *aquaticus* and *P*. *coxalis* , mainly the shape of the pleopod II was examined (identification key of Eiseler 2010). Depending on sampling success, we extracted the DNA of 1–16 specimens per site (Table S1). In 2019, DNA was extracted following the salt precipitation protocol as described by Weiss and Leese (2016). In 2020, pipetting steps of the extraction were conducted on a Biomek FX^P^ liquid handling workstation (Beckmann Coulter, Bread, CA, USA) using a modified version of the bead extraction protocol of the NucleoMag Tissue kit (Macherey‐Nagel, Düren, Germany) as described in Buchner et al. (2021). After the extraction, DNA concentration was measured with the Qubit dsDNA BR Assay Kit (Life Technologies; Thermo Fisher Scientific).

For all specimens, the barcoding fragment of the mitochondrial cytochrome c oxidase I (COI) gene was amplified with the standard primers HCO2198 and LCO1490 (Folmer et al. 1994). For the 2019 samples, we used the following PCR protocol: 0.2 μL VWR Taq‐polymerase (5 U/μL), 2.5 μL key buffer Mg^2+^ free (10×, VWR), 2.5 μL dNTPs (2 mM), 2.5 μL MgCl_2_ (25 mM), 0.125 μL of each primer (100 μM), 1–3 μL of DNA template, and filled up to 25 μL with PCR water. The COI fragment was amplified with the following settings: initial denaturation at 94°C for 2 min; 33–36 cycles of denaturation at 94°C for 20 s, annealing at 46°C for 30 s, and extension at 72°C for 60 s; final extension at 72°C for 5 min. Due to changes in the lab, the 2020 samples were amplified with a different protocol as follows: 5 μL Dream‐Taq^TM^ Hot Start Green PCR Master Mix (2×, Thermo Fisher Scientific, Waltham, MA, USA), 0.05 μL of each primer (100 μM), 0.5 μL of DNA template, filled up to 10 μL with PCR water. To increase primer specificity, amplification was performed using a touchdown PCR, where the annealing temperature was decreased by 1°C in each of the first 10 cycles: initial denaturation at 95°C for 3 min, 10 cycles of denaturation at 95°C for 30 s, annealing at 56°C–46°C for 30 s, extension at 72°C for 60 s, followed by 40 cycles with the same program with an annealing temperature of 46°C, and a final extension at 72°C for 5 min. Prior to sequencing, PCR products were purified in both years using 0.5 μL of ExoI (20 U/μL) and 1 μL of FastAP (1 U/μL, both Thermo Fisher Scientific, Schwerte, Germany). The reaction was incubated for 25 min at 37°C followed by an inactivation step at 85°C for 15 min. Bidirectional Sanger sequencing was performed at Eurofins Genomics.

Samples for ddRAD‐seq were chosen based on location, year, COI haplotype, and DNA concentration, aiming to analyze eight specimens per site and year, if enough individuals had been sampled. In total, we analyzed 280 individuals of both species on three sequencing lanes and therefore enhanced specimen numbers up to 12 specimens per site, where enough individuals had been sampled. This resulted in 164 specimens of *A*. *aquaticus* and 116 of *P*. *coxalis* . The ddRAD libraries were generated according to the protocol described in Hupało et al. (2023). Details of the sample preparation for each individual are given in Table S2. Depending on the initial DNA concentration, up to 600 ng DNA was used for double digestion with the FastDigest restriction enzymes *Csp6*I and *Pst*I (Thermo Fisher Scientific). For choosing most suitable restriction enzymes and calculating adapter quantities for ligation (Peterson et al. 2012), expected cut frequencies and number of fragments were estimated by *in silico* digestion using the script genomecut.pl. (Rozenberg, https://github.com/evoeco/radtools/). Now, a first draft reference genome would be available for *A*. *aquaticus* (Bakovic et al. 2021), but this was not available when designing the study. Therefore, we used the genomes of two other isopods ( *Armadillidium vulgare* and *Ligia exotica* with the NCBI accession numbers LYUU01000000 and BDMT000000000) for the estimation. This resulted in average cut frequencies of 547 bp for *Csp6*I and 7689 bp for *Pst*I. In the PCR, 16 cycles were sufficient for amplifying an adequate number of fragments for sequencing. After measurement, samples were pooled equimolarly into three libraries, aiming at 40 ng of DNA per specimen. The first lane contained 96 specimens of *A*. *aquaticus* , the second 68 *A*. *aquaticus*, and 29 *P*. *coxalis* , and the third 88 *P*. *coxalis* specimens. In addition, the third lane contained 8 ddRAD libraries of *Gammarus pulex* and *G*. *fossarum* from another study, resulting in 96 specimens per lane.

### 2.3 COI Data Analysis

The obtained COI sequences were assembled and edited in Geneious Prime 2022.0.2 (https://www.geneious.com) and sequences of each species were aligned with MAFFT 1.4.0 (Katoh and Standley 2013) as implemented in Geneious with default settings. To check species assignment, sequences were compared with the NCBI database (NCBI Resource Coordinators 2018). Sequences that were too short or where quality was not sufficient for haplotype determination were only used for species assignment but excluded from further analyses (Table S2). The alignments of the remaining sequences were cropped to the length of the shortest sequence per alignment. All following analyses were conducted similarly for both species. First, COI haplotype distances and their frequencies were calculated and visualized as minimum spanning networks (Bandelt, Forster, and Röhl 1999) in Popart v.1.7 (Leigh and Bryant 2015) and colored according to the catchment. To delimitate MOTUs which might represent different cryptic species, we used the ASAP approach (assemble species by automatic partitioning; Puillandre, Brouillet, and Achaz 2021) on the web server, applying the Kimura (K80 ts/tv 2.0) model for computing distances and using otherwise the default settings. For *A*. *aquaticus* , sequences of main haplotypes from ASAP groups were compared to the studies of Verovnik, Sket, and Trontelj (2005) and Sworobowicz et al. (2020) to find out to which group or potential cryptic species the specimens from the Emscher catchment belong. For *P*. *coxalis*, no such detailed European phylogeny was found and therefore ASAP group sequences were compared to sequences found in closest proximity to our sampling sites in the Word Asellidae database (WAD, https://gotit.univ‐lyon1.fr/en/), using the distribution data based on the study of Saclier et al. (2024) with names of the TH method for species identification.

To analyze temporal as well as spatial differences between sampling years and locations, haplotype diversity, nucleotide diversity, and pairwise *F*_ST_ values were calculated in Arlequin (v 3.5.2.2, Excoffier and Lischer 2010) for both years separately as well as combined. Here, only sites were included, where more than five specimens have been analyzed. For visualization, *F*_ST_ values and corresponding *p* values were plotted in heatmaps using RStudio (Posit team 2024) and the R packages reshape 2 (Wickham 2007) and ggplot2 (Wickham 2016). To evaluate if the population structure can be explained by geographic distances between sampling sites (isolation by distance, IBD), Mantel tests were conducted with the R‐package vegan (Oksanen et al. 2019) using *F*_ST_ values as genetic distances and waterway distances as geographic distances. Geographic distances were calculated using QGIS v.2.14.14 (http://qgis.org) with a stream layer provided by the federal state authority LANUV (Gewässerstationierungskarte des Landes NRW LANUV NRW (2013)). As only few sites were successfully sampled in the Berne catchment for both species, Mantel tests were also conducted using only sites from the Boye system, and both tests were plotted together in an IBD plot using the R graphics package (v.4.4.0; R Core Team 2024). Furthermore, maps for visualization of sampling sites and haplotype distribution were generated with the same stream layer as above in QGIS v.2.14.14 (http://qgis.org) and edited with Adobe illustrator 2024.

### 2.4 ddRAD‐Seq Data Analysis

Preprocessing of the three ddRAD‐seq libraries was performed similar to Weiss et al. (2018), including demultiplexing and removing PCR duplicates. To identify loci and genotypes, denovo_mao.pl. of Stacks v.1.34 (Catchen et al. 2013) was used. Similar to Hupało et al. (2023), the Stacks pipeline was run with eight different parameter settings according to the guidelines in Paris, Stevens, and Catchen (2017) to identify optimal parameter settings for each species. The following analyses were executed using the workflow management tool Snakemake (Köster and Rahmann 2012). The workflow contained stacks2fasta.pl. (Macher et al. 2015) and several R and python scripts for data reformatting, filtering, and population genetic analyses same as in Weiss et al. (2018). For the subsequent analyses, we used the following general filtering settings: maximum number of single nucleotide polymorphisms (SNPs) per locus was 12, of which only one was subsequently used; minor allele frequency of 1%, a locus had to be present in 90% of the individuals to remain in the dataset. Furthermore, specimens with > 40% missing data were excluded from final analyses. Evaluation of Stacks parameter settings was performed by calculating basic population statistics, such as observed heterozygosity (*H*_O_), observed gene diversity (*H*_S_), overall gene diversity (*H*_T_), and overall *F*_ST_ and *F*_IS_, which were calculated using hierfstat (Goudet 2005) in R v.4.4.0 (R Core Team 2024). To identify genetic clusters, principal component analyses (PCAs; Patterson, Price, and Reich 2006) were performed, and individual ancestry coefficients were estimated based on sparse nonnegative matrix factorization algorithms (sNMF; Frichot et al. 2014) using the R‐package LEA (Frichot and François 2015). In the sNMF analysis, the number of clusters varied between 1 and 15 with 40 replicates and 500,000 iterations per replicate. For selecting the most probable number of clusters (*K*), cross‐entropy values were compared. To analyze temporal and spatial differentiation, pairwise *F*_ST_ values (after Weir and Cockerham 1984) between sites (for both years separately and combined) were calculated, and significance was tested by bootstrapping over loci (1000 replicates, 0.025/0.975 confidence intervals) with the R‐package hierfstat using only sampling sites with > 5 specimens. To test for IBD, the same analyses as described for the COI data were conducted. The divMigrate function (Sundqvist et al. 2016) of the R‐package diveRsity (Keenan et al. 2013) was used to assess directional relative migration rates and to detect asymmetries in gene flow using the measure D (Jost 2008) for sites with > 5 specimens. Genetic diversity was estimated by calculating H_O_ and allelic richness (AR) in each population (for both years separately and combined) using the same R‐package. Furthermore, Neighbor‐Net networks (Bryant and Moulton 2004) were calculated using SplitsTree v. 4.14.5 (Huson and Bryant 2006).

## 3 Results

### 3.1 Population Structure of *Asellus aquaticus*

**FIGURE 2:**
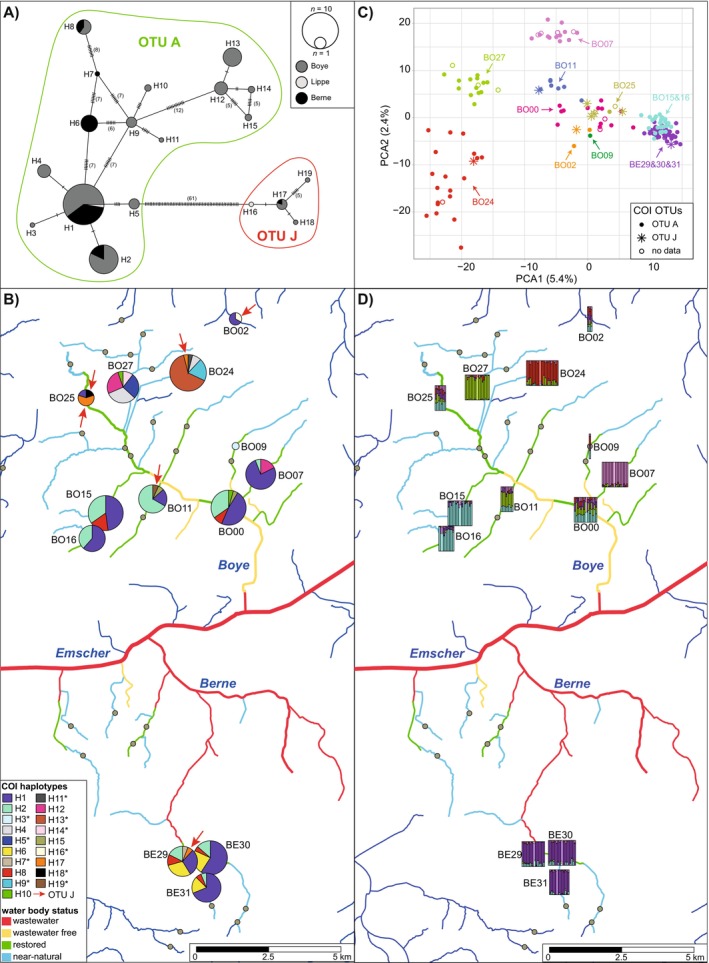
Population structure of *A*. *aquaticus* in the Emscher catchment. (A) COI minimum spanning network colored according to catchments. Vertical lines indicate mutations between haplotypes. (B) COI haplotype map showing the haplotype composition. The sizes of haplotype pie charts are scaled according to the number of sequences per site and haplotypes of OTU J are indicated by an arrow. (C) Principle component analysis of the ddRAD‐seq data. Individuals are colored according to location and different symbols are used for different COI OTUs. (D) Ancestry estimates from sNMF analysis for *k* = 5 displayed on the map, with vertical bars representing individual ancestry coefficients.

In total, we extracted DNA from 215 specimens of *A*. *aquaticus* , which were found at 13 of the 41 sampling sites, including nine sites at which they were detected in both years (Table S1). For all 215 specimens, the COI sequence data verified the species identification. However, some of the sequences were too short or quality was insufficient to determine haplotypes. The resulting final COI alignment included 199 sequences (Data S1), had a length of 562 bp, and contained 89 variable sites, of which one was a nonsynonymous substitution. In total, we detected 19 haplotypes (H1–H19), of which two had a frequency of > 15%, 14 had a frequency of < 5%, and 10 only occurred at one sampling site, each (Table S3). Most of these private haplotypes had a frequency of < 3% with the exception of H13 at site BO24 (8%). The partition with the best ASAP score indicated two potential cryptic species (Table S4), which could be assigned to the widely distributed OTU A (Sworobowicz et al. 2020; or “Central Europe” group in Verovnik, Sket, and Trontelj 2005) and OTU J (or “Trans‐Alpine” group), respectively. Most of the specimens from the Emscher catchment belonged to OTU A (15 haplotypes), while OTU J contained only four haplotypes (H16‐19) and was separated by at least 61 mutations (H5–H16) from OTU A (Figure 2A). Specimens with haplotypes from both OTUs were found in each of the three catchments (Figure 2A,B).

When considering the haplotype distribution in more detail (Figure 2B), a generally high haplotype diversity per site becomes evident. Haplotype diversity ranged between 0.404 and 0.824 with a mean of 0.616 and nucleotide diversity between 0.001 and 0.047 with a mean of 0.015 (Table S5). With respect to population structure, we found populations from near‐natural sites in the northern Boye catchment (BO24 and BO27) to be significantly differentiated from all other sites (Figure S1A), containing only private and rare (shared with other populations but frequency < 10%), but none of the main haplotypes. All other populations of the Boye catchment were significantly differentiated from some, but not all populations in the Boye and the Berne catchment. An IBD pattern was only detected when regarding solely distances within the Boye catchment (*r* = 0.563, *p* = 0.001), but not when including also distances to Berne populations (*r* = −0.008, *p* = 0.46; Figure S2A). Allele frequencies were stable through time, as indicated by the lack of significant differentiation between populations per individual sites across years (Figure S1A).

To investigate the population structure at higher resolution, we generated ddRAD data for 164 specimens. Depending on the Stacks settings, we obtained between 2444 and 2806 loci when including all specimens. As all basic population genetic statistics were similar between settings, we decided to further use the Stacks setting, which resulted in the highest number of loci (Table S6). Excluding specimens with > 40% missing data (four specimens) resulted in 3302 loci, which were used for all further analyses. To get a first overview of the population structure and check if the ASAP COI OTUs represent cryptic species, we conducted a PCA. Here, the first 12 axes were significant, with the first three explaining 5.4%, 2.4%, and 1.8% of the variation, respectively. As visible in Figure 2C, most of the individuals from one sampling site clustered closely together regardless of the corresponding haplotype group, indicating the presence of only one species in the area. This was also supported by the Neighbor Net, where one big group was visible with specimens from the same site clustering mostly together (Figure S3). In addition to the PCA, the fine‐scale population structure is also visible in the sNMF analysis, where five clusters best represent the population structure according to the cross‐entropy criterion (Figure S4B). When displaying the sNMF plot for *k* = 5 on the map (Figure 2D), five separated populations are visible. Here, two of the populations from the northern near‐natural sites of the Boye catchment (BO24 and BO27) are each separated from all other populations (Figure 2C), also corresponding with the PCA analysis. Similarly differentiated from all is population BO07, which is located in a restored stream further downstream in the Boye catchment. Another cluster is mainly present in populations BO15 and BO16, both located in close proximity (1 km distance) in the same stream. In the PCA, these two populations are not distinguishable from the three Berne populations regarding the first two PCA axes (Figure 2C) but are separated by the third axis (Figure S4A). In the sNMF analysis, the three Berne populations (BE29, BE30, and BE31) form the fifth cluster. The remaining five populations, including BO02 which was located in a neighboring catchment, were a mix of all five clusters, but in different proportions, also visible in the PCA where they grouped together. Nevertheless, nearly all *F*_ST_ values indicated a significant differentiation in a range between 0.006 and 0.189 (considering only comparisons for *n* > 5), except comparisons between BE30 and both, BE29 and BE31, but including the comparison of BE29 and BE31 (Figure S1B). Furthermore, low *F*_ST_ values were found between the Berne and some Boye populations (i.e., BO16, BO15, BO00, and BO25). Similar to the COI data, an IBD pattern was only found when considering solely comparisons within the Boye catchment (*r* = 0.4, *p* = 0.031; Figure S2C) because in some cases, differentiation was lower between populations located in different catchments, than within the Boye catchment. When comparing specimens sampled at the same location but in different years, none of the comparisons indicated a significant differentiation. Highest gene flow was detected between the three Berne populations and between BO15 and BO16 in the DivMigrate analysis (Figure S5A). Furthermore, none of the pairwise comparisons of directional gene flow between sites was significantly asymmetric. Genetic diversity in terms of allelic richness ranged between 1.38 and 1.47 (mean: 1.43), while observed heterozygosity was relatively constant between sites, ranging between 0.11 and 0.13 (mean: 0.12; Table S5).

### 3.2 Population Structure of *Proasellus coxalis*

**FIGURE 3:**
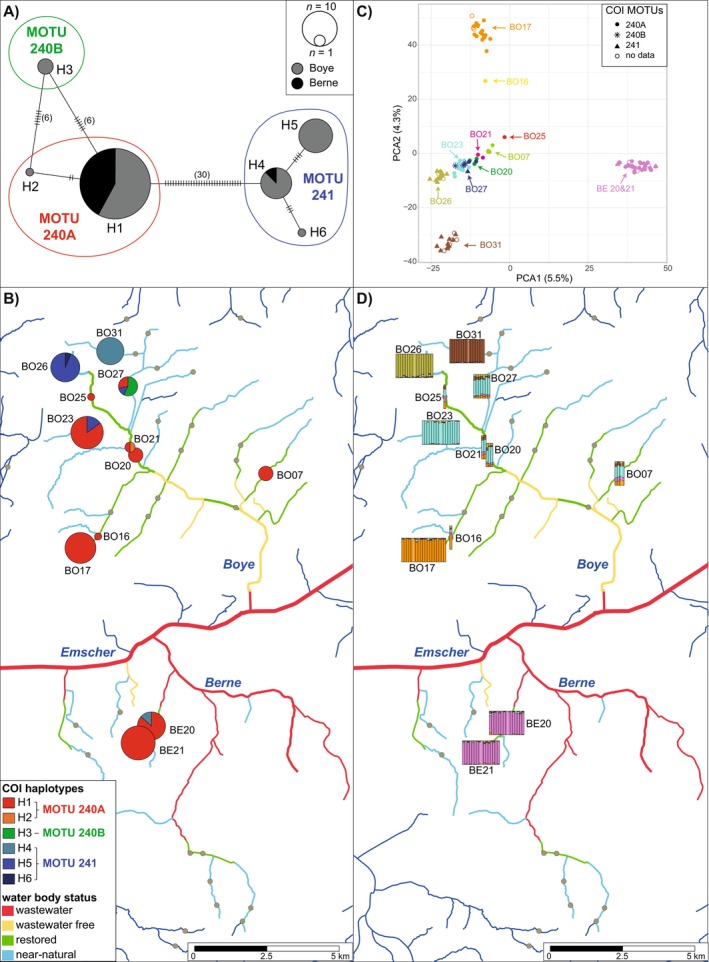
Population structure of *P*. *coxalis* in the Emscher catchment. (A) COI minimum spanning network colored according to catchments. Vertical lines indicate mutations between haplotypes. (B) COI haplotype map showing the haplotype composition. The sizes of haplotype pie charts are scaled according to the number of sequences per site. (C) Principle component analysis of the ddRAD‐seq data. Individuals are colored according to location and different symbols are used for different COI OTUs. (D) Ancestry estimates from sNMF analysis for *k* = 5 displayed on the map, with vertical bars representing individual ancestry coefficients.

*P*. *coxalis* was detected at 12 sites, including five sites at which specimens of the species were only found in 1 year (Table S1). At the four sites, where both species occurred in syntopy, *P*. *coxalis* was always less frequent than *A*. *aquaticus* . In total, we extracted DNA for 142 specimens and could use all COI sequences to verify the morphological species identification. Due to insufficient quality, some of the sequences were excluded from the final analyses, resulting in 124 sequences in the final alignment. The final alignment had a length of 520 bp and contained 40 variable sites of which three were nonsynonymous substitutions (Data S2). In contrast to *A*. *aquaticus* , only six different haplotypes (H1–H6) were detected, of which three had a frequency of < 5% and were private for different sampling sites (Table S7). The best two partitions of the ASAP approach indicated three potential cryptic species, of which two were assigned to MOTU 240 (named MOTU 240A and 240B hereafter), and the third was assigned to MOTU 241 (both names from WAD, HT approach, Saclier et al. 2024). Most of the specimens belonged to MOTU 240A, which contained the haplotypes H1 and H2 and was present in both Boye and Berne catchments (Figure 3A). MOTU 240B only consisted of haplotype H3 (four specimens, site BO27). MOTU 240A and B were separated by six mutations, while MOTU 241 was separated by 30 mutations from MOTU 240A and 36 from MOTU 240B. MOTU 241 consisted of haplotypes H4–H6 and was present in 29% of the specimens mainly in the Boye system, but H4 also occurred at Site BE20 (Figure 3B). Similar to *A*. *aquaticus* , populations in the north of the Boye system (BO26, BO27, and BO31) were significantly differentiated from all other populations (Figure S1C), while Berne populations were not significantly differentiated from the southern Boye populations. At five sites, less than five individuals were found and these populations were therefore excluded from the *F*_ST_ analysis. For both the full dataset and the Boye catchment alone, no IBD pattern was detected (Figure S2B). When considering only the Boye catchment, a similar trend as for *A*. *aquaticus* was visible, but too few comparisons remained to detect a significant correlation (*r* = 0.441, *p* = 0.133). Similar to *A*. *aquaticus* , no temporal differentiation was detected in the COI data (Figure S1C). Genetic diversity was lower in comparison to *A*. *aquaticus* with a mean haplotype of 0.187 and a mean nucleotide diversity of 0.008 (Table S5). Haplotype diversity ranged between 0 and 0.268 with the exception of Site BO27 where both species occurred together and where highest haplotype (0.667) and nucleotide diversity (0.0238) were found.

To analyze fine‐scale population structure, ddRAD libraries were generated for 116 specimens. Depending on the Stacks settings, 9848–11,764 loci were retained (Table S6) when all specimens were included. Because all basic population genetic statistics were similar between settings, we decided to use the stacks setting with the most loci (“m3 M3 N5 n4”) for the final analysis. Excluding specimens with > 40% missing data (one specimen) resulted in 12,186 loci which were used for all further analyses. To check if the ASAP COI MOTUs represent cryptic species and analyze the population structure, we conducted a PCA. The first nine axes were significant, with the first three explaining 5.5%, 4.3%, and 4.1% of the variation, respectively. When plotting the first to axes (Figure 3C), clustering was not driven by the haplotype group, but by the sampling site, as all specimens from one sampling site clustered together regardless of the ASAP group. This was also visible in the Neighbor Net, where specimens from most of the sites were clearly separated, but not structured according to the haplotype groups (Figure S6). The generally strong population structure is also visible in the sNMF analysis, where five clusters best represented the population structure according to the cross‐entropy criterion (Figure S4C). On the map, five clearly separated groups are visible (Figure 3D). Similar to *A*. *aquaticus* , two populations from the northernmost near‐natural streams, that is, BO26 and BO31, were clearly separated from all other populations. However, this does not include BO27, which was strongly differentiated for *A*. *aquaticus* , but clusters together with a big group of more downstream populations (BO23, BO20, BO21, BO25, and BO07) for *P*. *coxalis* . The same is true for the syntopic population BO07, which was differentiated from *A*. *aquaticus* , but belonged to the main cluster of *P*. *coxalis* . For both species, BO25 was not as differentiated as the other near‐natural upstream populations. For *P*. *coxalis* , only one specimen was found at this site, which was, however, clearly differentiated from the close upstream Site BO26 (1.87 km). Similar to *A*. *aquaticus* , specimens from the oldest restored sites in the Vorthbach (BO17 and BO16 for *P*. *coxalis* ) formed a distinct cluster. However, the single specimen found at BO16 (0.3 km downstream of BO17) was separated from BO17 in the PCA. The last cluster is formed by individuals from the Berne catchment (BE20 and BE21), which were, in contrast to indications from the COI analysis, strongly isolated. The general strong small‐scale differentiation between sampling sites is also indicated by the F_ST_ values, which were all significant (considering only comparisons for *n* > 5) and ranged from 0.003 to 0.197 (Figure S1D). While most of the populations from the big group from the sNMF analysis were too small to reliably calculate *F*_ST_ values, the included comparison between BO23 and BO27 also indicated significant differentiation, but showed the second lowest value (0.093) after the comparison of the two Berne populations (BE20 and BE21; 0.003). In contrast to the COI data and both data sets for *A*. *aquaticus* , an IBD pattern for the whole dataset was detected for *P*. *coxalis* (*r* = 0.558, *p* = 0.003; Figure S2D). When considering only distances from the Boye catchment, a similar but non‐significant trend was found similar to the COI data (*r* = 0.408, *p* = 0.2). In contrast to *A*. *aquaticus* , low but significant differentiation was indicated for three of the temporal comparisons (BE21, BO17, and BO26), while the others were not significantly differentiated (Figure S1D). Highest gene flow was detected between the two Berne populations in the DivMigrate analysis (Figure S5B) but was low between all other populations (only comparisons for *n* > 5). Furthermore, no significantly asymmetric gene flow was detected. Allelic richness was similar to *A*. *aquaticus* , ranging from 1.4 to 1.48 (mean: 1.44), while observed heterozygosity was similar between all sites, ranging between 0.13 and 0.14 (mean: 0.14; Table S5).

## 4 Discussion

In this study, we used a population genomic approach to quantify patterns of genetic diversity and population structure of two pollution‐tolerant isopod species with different postglacial recolonization histories in a restored river system in a heavily urbanized area. Our first hypothesis postulated that *A*. *aquaticus* will show a high genetic diversity as well as a high connectivity between sampling sites following an IBD pattern. However, while we found a high COI haplotype and nucleotide diversity, nuclear genetic diversity was not particularly high, and connectivity between most of the sampling sites was lower than expected according to the hypothesis.

As presumed by Zizka, Weiss, and Leese (2020), we found both OTU A and OTU J in the Emscher catchment. Otherwise, OTU J had only been recorded in Southern Germany, France, Italy, Slovenia, and Croatia (“Trans‐Alpine” Group), but sometimes in sympatry with OTU A, which had the widest distribution range of all discovered OTUs (Sworobowicz et al. 2015). Additionally to the mitochondrial COI gene, Sworobowicz et al. (2015) analyzed the less variable nuclear 28S rDNA gene, finding several mitonuclear discordance patterns including OTUs A and J, which shared two 28S haplotypes, while other OTUs were reciprocally monophyletic. This pattern was interpreted primarily as a result of incomplete sorting of the slower‐evolving nuclear lineages, or in some cases, as a result of introgression of formerly isolated peripatric mitochondrial lineages. Our highly resolved nuclear SNP data reject that OTUs A and J represent distinct species with incomplete nuclear lineage sorting. Instead, our data agree with the results of the slowly evolving 28S gene in Sworobowicz et al. (2015) and suggest that both OTUs represent one interbreeding species and might have originated via past isolation. The results suggest that in case of *A*. *aquaticus* , COI may greatly overestimate species diversity if OTUs are interpreted as species. An extremely high intraspecific diversity of the COI gene has been found to be a general pattern for terrestrial and freshwater isopods and interpreted to be the result of phylogeographic events, *Wolbachia* infections, atypical mitochondrial DNAs, heteroplasmy, or a combination of these factors, instead of an indication for the presence of multiple cryptic species (Raupach, Rulik, and Spelda 2022). In our study, specimens of the less frequent OTU J always occurred in sympatry with OTU A and were not differentiated in the nuclear SNP data, but clustered together with the other specimens from the same sampling site. As the distribution of OTU J is otherwise transalpine and the COI diversity was much lower in the area than of the dominant lineage OTU A, it can be assumed that OTU J colonized the Emscher area only later from Southern Europe. Both OTUs have also been identified in a metabarcoding study (Zizka, Weiss, and Leese 2020), indicating that such an approach is suitable for detecting haplotype diversity.

While we found a relatively high genetic diversity with regard to mitochondrial COI haplotype and nucleotide diversity, nuclear diversity in terms of allelic richness (AR) was not particularly high and H_O_ was relatively low at all sampling sites. AR was comparable to other macroinvertebrate species in a less degraded, but strongly fragmented area relatively close to the Emscher catchment (Ruhr catchment; Weiss, Weigand, and Leese 2022). The lower nuclear diversity is more in accordance with the general strong, but unexpected population structure. Because *A*. *aquaticus* is relatively pollution tolerant, was present in the streams directly after restoration, and might even have survived harsh conditions in polluted waters (Gillmann, Hering, and Lorenz 2023), we expected high connectivity between sites and accordingly a low differentiation. At a few neighboring sites, for example, at sites in the Berne system, where distance between sites was < 1.8 km, high gene flow was detected in accordance with the hypothesis. However, at the neighboring sites in the Boye system (i.e., BO15 and BO16, 1 km apart), gene flow was already reduced, even though it was still high in comparison to all other sites within the Boye system, which had a distance of 1.9–10.5 km from each other. In accordance with an IBD pattern, populations within the Boye catchment were significantly differentiated by distance. However, this was not the case when populations of the Berne catchment were included because differentiation between Berne and Boye populations was sometimes lower and estimated migration rates were higher than within the Boye catchment over shorter waterway distances. The lowest differentiation of Berne populations was found to Vorthbach (BO15 and BO16) and Boye (BO00 and BO25) populations. However, in‐stream migration through Berne, Emscher, and Boye is unlikely because of the distance and the many still existing migration barriers like wastewater, channelization, and water‐level differences between Boye and Emscher. Therefore, passive over land dispersal by human activities (e.g., river restorations or monitoring at different sites in the same timeframe) or bird‐mediated transport, that is, by specimens being carried in the feathers and/or weeds associated with aquatic birds (Coughlan et al. 2017; Sworobowicz et al. 2020; Verovnik, Sket, and Trontelj 2005), seems to be the more likely explanation here.

In contrast, differentiation between Boye populations was in some cases higher than expected considering the distance between sites, indicating that other additional factors are shaping the genetic structure. Here, two of the populations in the near‐natural sites (BO27 and BO24) and three populations from restored sites (BO07, and BO15 and BO16, together), each had their own cluster in the sNMF analysis, indicating the presence of migration or gene flow barriers and that restored sites were either recolonized after restoration from an unidentified source or that populations have survived in the polluted parts and remained isolated there. In the region, many small ponds containing *A*. *aquaticus* populations exist, which were not sampled here, but from which recolonization of stream habitats by passive dispersal via birds could have happened. To analyze the role of this dispersal mechanism in structuring metapopulations, it would be important to also genetically analyze these populations. For two of the populations, BO24 and BO07, potential migration barriers exist, while they were less obvious for the other isolated populations. At the outlet of the Nattbach (BO07) into the Boye, an impassable drop existed, which was only recently (2021) removed. Furthermore, the lower part of the Quaelingsbach (BO24) is still channelized as it runs directly next to a highway and the middle part falls often dry in summer. For BO27 and BO15/16, no direct barrier could be identified, but also here, parts of the streams sometimes fall dry in summer and most parts of the Boye were only restored relatively recently. Another potential migration barrier, a pumping station where the water of the Boye is pumped through a pipe because of mining subsidence, exists in the Boye downstream of site BO25. However, this population was not as isolated as would be expected, but clustered together with other populations from restored sites, indicating again the importance of passive dispersal. The absence of obvious migration barriers for some populations and the isolation of other populations despite the possibility of passive dispersal can indicate that in some cases gene flow is restricted despite possible migration. This can occur when specimens disperse but cannot establish in the new population, which can be explained by either a density‐dependent priority effect (Fraser, Banks, and Waters 2014), or according to the monopolization hypothesis by a combination of numerical advantage together with adaptation of the first migrants (de Meester et al. 2002). Here, it might be possible that the individuals from the near‐natural sites (BO24 and BO27) are less adapted to the conditions in the restored sites than other source populations, which might even have survived in conditions prior to restoration. This could lead to a priority effect of source populations from formerly polluted sites over populations from restored sites. Priority effects and isolation by adaptation have also been identified in other studies for different species (e.g., Funk, Egan, and Nosil 2011; Nosil, Funk, and Ortiz‐Barrientos 2009; Nosil, Egan, and Funk 2008; Urban and De Meester 2009). However, we could not test this hypothesis with our data.

One additional factor, which could have shaped the population apart from distance, migration barriers, and adaptation, is competition either between *P*. *coxalis* and *A*. *aquaticus* or with the two amphipod species, *G*. *pulex* and *G*. *fossarum*, which co‐occur in the system at many sites and have a similar ecological niche (Graça, Maltby, and Calow 1994a). For the two isopod species, it is difficult to predict which of the species has a competitive advantage because both species were found to have an advantage over the other in different studies (e.g., Burmeister 2003; Costantini and Rossi 1998), or showed potential for niche differentiation when occurring in sympatry (Costantini et al. 2005; Rossi, Basset, and Nobile 1983), so competitive relationship could be different according to locality or environmental conditions (Kemp et al. 2020). In our study, we found less *P*. *coxalis* than *A*. *aquaticus* specimens and at the four sites, where both species occurred, we always found more *A*. *aquaticus* specimens. However, at most of the sites, only one of the species was found. Here, *A*. *aquaticus* was mainly found at permanent sites, while *P*. *coxalis* was found at temporary sites which often dry out in summer or directly below drying stream sections, indicating that *P*. *coxalis* can better cope with these conditions. When comparing *G*. *pulex* with *A*. *aquaticus* , it was found that *G*. *pulex* normally dominates in clean water, while in more polluted water, *A*. *aquaticus* becomes the dominant species (MacNeil et al. 2002; Whitehurst 1991). Similar observations have also been made in the Emscher catchment, where over time a decrease of *A*. *aquaticus* was found together with an increase in *G*. *pulex* (Gillmann, Hering, and Lorenz 2023). Here, we found co‐occurrence of both isopod species and either one or both of the amphipod species at all but one site (BO24). Therefore, it is difficult to understand how the presence of amphipods could have shaped the population structure in the two isopod species, but as water quality should improve more over time, it could be expected that populations of isopod species will further decline. However, it has not been found, yet, what drives the competitive advantage of *G*. *pulex* over *A*. *aquaticus* in cleaner water, except that *G*. *pulex* is more adapted to higher flow velocities. Until now, there has been no evidence of competition for spatial resources (Graça, Maltby, and Calow 1994b) or an indication that food is important in the separation of both species (Graça, Maltby, and Calow 1994a). Therefore, continuing coexistence of the four species could still be possible, and co‐occurrence and competition dynamics would need to be addressed, for example, in a time‐series study.

Our second hypothesis postulated genetic diversity to be lower in the similarly pollution‐tolerant species *P*. *coxalis* , especially in the COI gene, because it is an alien species, but connectivity to be similarly high as in *A*. *aquaticus* . In accordance with the hypothesis, we found a much lower haplotype diversity in general (6 vs. 19 haplotypes), but also at all but one of the sampling sites. This can be expected in invasive species, when the number of initial colonists is small, leading to reduced genetic variation in comparison to source populations due to the genetic drift acting on founder populations (Sakai et al. 2001). However, when multiple introductions occur from different source populations, genetic diversity can also by higher than in any single source population (Sakai et al. 2001). In contrast to haplotype diversity, AR and *H*_O_ were not reduced in comparison to *A*. *aquaticus* , but even slightly higher. To better understand the colonization process of *P*. *coxalis* from southern Europe, it would be essential to analyze both mitochondrial and nuclear markers in the native distribution range as well as along proposed migration routes. The integration of nuclear markers is especially advisable here because similar to *A*. *aquaticus* , three OTUs or potential cryptic species were identified for *P*. *coxalis* with the COI gene, and many more were found and deposited in the World Asellidae Database (WAD) by different sources and delimitation approaches (Eme et al. 2018; Morvan et al. 2013; Saclier et al. 2024). In Zizka, Weiss, and Leese (2020), *P*. *coxalis* was only detected at one site in the Emscher catchment and all haplotypes belonged to MOTU 240, which was also the dominant MOTU, when combining ABGD groups 240A and 240B in our study as well as when considering distributions in WAD. Here, MOTU 240 had the broadest distribution, ranging from Spain over France and Italy to Croatia, and Bosnia and Herzegovina, with occurrences also in Germany, the Netherlands, and Sweden. Even though less abundant, the other OTU detected in the Emscher region, MOTU 241, had a similar southern distribution range from the south of France to Bosnia and Herzegovina with the northernmost occurrence in the east of France close to the German border. Both MOTUs have been found to occur in sympatry. As with OTUs in *A*. *aquaticus* , our data clearly show evidence for only one species in our study area, suggesting also that these MOTUs might, in general, belong to one species. To delimit species in the broad *P*. *coxalis* species group, which also includes many subspecies, a Europe‐wide sampling would be needed. However, our data show how important it would be to integrate nuclear data here to disentangle species in this species complex and to understand invasion routes.

As for *A*. *aquaticus* , we expected high levels of connectivity within Boye and Berne systems. However, estimated migration rates between sites were even lower than in *A*. *aquaticus* except for the two neighboring sites in the Berne system, which were only 0.8 km apart, but together clearly separated from populations of the Boye catchment. While this stream still contains wastewater in the downstream part, it can be temporarily dry in summer in the restored part, which could explain why we found low but significant differentiation between time points. Furthermore, there are small ponds located close by, from which recolonization after restoration and also after dry periods can happen, for example, via aquatic birds. Another explanation for recolonization after restoration could be passive long‐distance dispersal from more distant regions as has been suggested also, for example, for *A*. *aquaticus* (Sworobowicz et al. 2020, 2015). Also, the other sites, where we found differentiation between time points (BO17 and BO26) dry often out in summer, explaining the temporary instability and also partly the isolation of the populations. BO17 is close to the spring of the Vorthbach, where *P*. *coxalis* can probably survive the dry periods. While otherwise, no obvious migration barriers exist further downstream, for both of the other isolated populations in temporary streams (BO26 and BO31), an additional barrier (pumping station) exists downstream of the sites, explaining especially the strong differentiation between BO31 and BO27 detected by both marker systems. The other populations that were located closer to or within the Boye were less differentiated, belonging mainly to one genetic cluster, including also the one specimen from BO25. This site is located upstream of the Boye pumping station, but the specimen was closely related to the downstream populations BO20/BO21 than to the upstream population BO26, indicating passive dispersal events similar to *A*. *aquaticus* . In contrast to *A*. *aquaticus* , populations at both BO07 and BO27 were not as isolated for *P*. *coxalis* . At Site BO27, this indicates again that *P*. *coxalis* can cope better with the summer droughts, maintaining population connectivity among neighboring sites (i.e., BO23 and BO20/BO21). However, it is unlikely that *P*. *coxalis* can actively overcome the instream barrier that separates BO07 from the Boye, indicating passive dispersal mediated by humans or birds.

In contrast to *A*. *aquaticus* , genetic differentiation in *P*. *coxalis* was correlated with geographic distance for the whole sampling area, indicating together with the other analyses that Emscher and Berne represent strong migration barriers and that passive dispersal probably only plays a role at a more local scale for this species. However, like in *A*. *aquaticus*, genetic differentiation was in some cases higher than expected based on distance alone. The similarities between differentiation patterns in both species, even though they only co‐occurred at four sites, suggest that similar migration or gene flow barriers lead to the isolation of populations as discussed for *A*. *aquaticus* . The magnitude of differentiation observed between the different populations is expected if genetic drift (e.g., founder event by few individuals) or selective effects (e.g., priority effects) acted across the past three decades of recolonization after extinction (e.g., Montero‐Pau, Gómez, and Serra 2018; Weiss, Weigand, and Leese 2022; Angst et al. 2022).

## 5 Conclusions

Against our expectations, we found a strong small‐scale population structure within both species. This underlines the analytical power and importance of using high‐resolution genetic markers to analyze metapopulations and to identify potential barriers to migration or gene flow. While we could identify some migration barriers and found indications for passive dispersal probably by either birds or humans, further studies are needed to disentangle how effects of isolation‐by‐dispersal limitations, local adaptation, and biotic interactions shape metapopulation structure. Our study shows that a high local macroinvertebrate population genetic diversity has been maintained in the Emscher system, thus pointing to a high conservation value even in urban streams. Finally, we could also show the importance of integrating nuclear markers into species delimitation, especially in isopod species, where an extremely high COI diversity can be found within species. Here, we showed that both OTU A and J in *A*. *aquaticus* , and MOTU 240 and 241 in *P*. *coxalis* , represent only one species each in the sampling area. While final assessment should include more regions, the sympatric occurrence of both COI groups in each of the species suggests that they could generally be considered as one species in their distribution range.

## Author Contributions

**Martina Weiss:** conceptualization (equal), data curation (lead), formal analysis (lead), funding acquisition (supporting), investigation (lead), methodology (lead), project administration (lead), software (equal), visualization (lead), writing – original draft (lead), writing – review and editing (lead). **Armin W. Lorenz:** conceptualization (supporting), investigation (supporting), writing – review and editing (supporting). **Christian K. Feld:** investigation (supporting), writing – review and editing (supporting). **Florian Leese:** conceptualization (equal), data curation (supporting), funding acquisition (lead), investigation (supporting), project administration (lead), writing – original draft (supporting), writing – review and editing (supporting).

## Conflicts of Interest

The authors declare no conflicts of interest.

## Supporting information


**Table S1.** Sampling sites with coordinates (WGS84), sampling dates for both years, stream name, catchment affiliation, and ecological state. Furthermore, the number of specimens analyzed per genetic marker is given (in brackets) together with the number of specimens in the final analysis for each sampling site.

**Table S2.** Information for ddRAD library preparation per sample for *A*. *aquaticus* and *P*. *coxalis* , number of missing loci in final data set, indication if used in final analysis after filtering, COI haplotype, and NCBI accession numbers. Below, also haplotype information on specimens is given, for which only COI sequences were generated.

**Table S3.** Haplotype distribution for *A*. *aquaticus* . Given are numbers for each year and for both years together (indicated with gray background).

**Table S4.** Results for the best partitions found by ASAP using K80 as a substitution model for *A*. *aquaticus* and *P*. *coxalis* .

**Table S5.** Different measures for genetic diversity for both species per sampling site. Diversity measures for ddRAD are as follows: allelic richness (AR) and observed heterozygosity (*H*_O_). Measures for COI are haplotype diversity (HDiv) and nucleotide diversity (NDiv). *n* is the number of specimens for each marker. Diversity was only calculated for sites with *n* > 5.

**Table S6.** Summary statistics for all stacks settings for *A*. *aquaticus* and *P*. *coxalis* (Test) and for the final dataset (Final); loci limit = percentage of specimens required to have the loci, ma = minor allele frequency, *H*_O_ = observed heterozygosity, *H*_S_ = within‐population gene diversity, *H*_T_ = overall gene diversity, and best *K*: *K* with lowest cross‐entropy (median from all repetitions) in sNMF analysis.

**Table S7.** Haplotype distribution for *P*. *coxalis* . Given are numbers for each year and for both years together (indicated with gray background).

**Figure S1.** F_ST_ heatmaps for pairwise comparisons between sampling sites for *A*. *aquaticus* (A, B) and *P*. *coxalis* (C, D) and COI (A, C) and ddRAD data (B, D), respectively. Above the diagonal pairwise *F*_ST_ values are given and below either *p* values (COI data sets; values < 0.05 indicate significant differentiation) or the lower confidence interval (ddRAD data set; values > 0 indicate significant differentiation) is given. In the diagonal, *F*_ST_ values for the comparison between the years 2019 and 2020 are given, with a white square, when only samples from 1 year were available. Significant *F*_ST_ values are indicated in bold and all values are colored according to the level of differentiation.

**Figure S2.** Correlation between pairwise genetic distances (*F*_ST_; A and B COI data, C and D ddRAD data) and waterway distances for *A*. *aquaticus* (A, C) and *P*. *coxalis* (B, D).

**Figure S3.** Neighbor Net of *A*. *aquaticus* for the ddRAD data set. Branches are colored according to sampling sites.

**Figure S4.** PCA of the ddRAD data of (A) *A*. *aquaticus* and (D) *P*. *coxalis* , with the first plot showing the first and the second axes and the second plot showing the first and the third axes, respectively. (B, C) Show standard boxplots of cross‐entropy values (40 repeats) of sNMF analysis for final ddRAD datasets for *A*. *aquaticus* and *P*. *coxalis* , respectively.

**Figure S5.** Relative migration networks (D) for (A) *A*. *aquaticus* and (B) *P*. *coxalis* . Only populations with > 5 individuals were included in the analysis.

**Figure S6.** Neighbor Net of *P*. *coxalis* for the ddRAD data set. Branches are colored according to sampling sites.

**Data S1.** Final COI sequence alignment of *A*. *aquaticus* .
**Data S2**. Final COI sequence alignment of *P*. *coxalis* .

## Data Availability

The data that support the findings of this study are available under the NCBI BioProject PRJNA1163041. Additionally, demultiplexed ddRAD data used for Stacks clustering are uploaded as BioSamples, and individual accession numbers are given in Table S2. COI haplotype sequences are available under the NCBI accession numbers PQ285564–PQ285588, indicated in Table S2.
