# Supplementary material for: Strong Small‐Scale Differentiation but No Cryptic Species Within the Two Isopod Species Asellus aquaticus and Proasellus coxalis in a Restored Urban River System (Emscher, Germany)
Source: Ecol Evol. 2024 Nov 18;14(11):e70575. doi: 10.1002/ece3.70575 (PMC11573423; doi:10.1002/ece3.70575)
Supplement: Supplementary file 1 — Table S1. Sampling sites with coordinates (WGS84), sampling dates for both years, stream name, catchment affiliation, and ecological state. Furthermore, the number of specimens analyzed per genetic marker is given (in brackets) together with the number of specimens in the final analysis for each sampling site. [file ECE3-14-e70575-s012.pdf]

Tab. S1: Sampling sites with coordinates (WGS84), sampling dates for both years, stream name, catchment affiliation, and ecological state. Further, the number of specimens analyzed per genetic marker is given (in brackets) together with the number of specimens in the final analysis for each sampling site.

Site	coordinates		Sampling date		Stream name	Catchment	Ecological state	*Proasellus coxalis*				*Asellus aquaticus*			
	Latitude	Longitude	2019	2020				COI 2019	ddRAD 2019	COI 2020	ddRAD 2020	COI 2019	ddRAD 2019	COI 2020	ddRAD 2020
**BO00**	51,544942	6,982729	05.04.19	21.04.20	Boye	Boye (Emscher)	restored 2019	0	0	0	0	8 (10)	8 (9)	15 (15)	7 (9)
**BO02**	51,603472	6,985075	08.04.19	16.04.20	Mühlenbach	Lippe	near-natural	0	0	0	0	0	0	3 (3)	3 (3)
**BO07**	51,555484	6,997598	02.04.19	21.04.20	Nattbach	Boye (Emscher)	restored 2011	3 (3)	3 (3)	1 (1)	1 (1)	4 (8)	8 (8)	13 (13)	9 (9)
**BO09**	51,564201	6,985123	02.04.19	21.04.20	Wittringer Mühlenbach	Boye (Emscher)	restored 2010	0	0	0	0	1 (1)	1 (1)	0	0
**BO11**	51,547875	6,943973	08.04.19	21.04.20	Kirchschemmsbach	Boye (Emscher)	restored 2007	0	0	0	0	0	0	15 (15)	8 (8)
**BO15**	51,543524	6,920588	29.03.19	22.04.20	Vorthbach	Boye (Emscher)	restored 2011	0	0	0	0	9 (9)	8 (8)	14 (14)	8 (8)
**BO16**	51,535679	6,913837	29.03.19	22.04.20	Vorthbach	Boye (Emscher)	restored 1993	0	0	1 (1)	1 (1)	2 (2)	2 (2)	11 (11)	8 (8)
**BO17**	51,534091	6,910535	29.03.19	22.04.20	Vorthbach	Boye (Emscher)	restored 1993	5 (6)	5 (5)	13 (15)	14 (14)	0	0	0	0
**BO20**	51,561190	6,932998	08.04.19	20.04.20	Boye	Boye (Emscher)	restored 2002	4 (4)	3 (3)	0	0	0	0	0	0
**BO21**	51,563604	6,930191	29.03.19	20.04.20	Boye	Boye (Emscher)	restored 2002	0	0	2 (2)	2 (2)	0	0	0	0
**BO23**	51,568118	6,908860	01.04.19	20.04.20	Schöttelbach	Boye (Emscher)	near-natural	8 (9)	8 (8)	12 (12)	8 (8)	0	0	0	0
**BO24**	51,586814	6,961502	01.04.19	20.04.20	Quaelingsbach	Boye (Emscher)	near-natural	0	0	0	0	9 (10)	9 (9)	16 (16)	12 (12)
**BO25**	51,579125	6,910851	08.04.19	20.04.20	Boye	Boye (Emscher)	restored 2009	0	0	1 (1)	1 (1)	0	0	5 (7)	7 (7)
**BO26**	51,589471	6,903014	04.04.19	16.04.20	Boye	Boye (Emscher)	near-natural	7 (9)	8 (8)	9 (12)	8 (8)	0	0	0	0
**BO27**	51,582391	6,929193	04.04.19	16.04.20	Brabecker Mühlenbach	Boye (Emscher)	near-natural	0	0	7 (7)	7 (7)	8 (10)	8 (9)	11 (12)	8 (8)
**BO31**	51,593251	6,920382	05.04.19	16.04.20	Wiesentalbach	Boye (Emscher)	near-natural	7 (8)	8 (8)	7 (12)	7 (8)	0	0	0	0
**BE20**	51,477440	6,940806	19.03.19	15.04.20	Pausmühlenbach	Berne (Emscher)	restored 2013	9 (10)	8 (8)	6 (7)	7 (7)	0	0	0	0
**BE21**	51,472198	6,934231	25.03.19	15.04.20	Pausmühlenbach	Berne (Emscher)	restored 2013	9 (10)	8 (8)	13 (13)	8 (8)	0	0	0	0
**BE29**	51,438118	6,960072	21.03.19	27.04.20	Borbecker Mühlenbach	Berne (Emscher)	restored 2011	0	0	0	0	10 (10)	8 (8)	7 (7)	7 (7)
**BE30**	51,437993	6,966351	21.03.19	27.04.20	Borbecker Mühlenbach	Berne (Emscher)	restored 2011	0	0	0	0	9 (10)	9 (9)	13 (14)	9 (9)
**BE31**	51,429386	6,969942	22.03.19	27.04.20	Kesselbach	Berne (Emscher)	near-natural	0	0	0	0	3 (5)	5 (5)	13 (13)	8 (8)
BO03	51,603125	6,928509	08.04.19	16.04.20	Schölsbach	Lippe	near-natural	0	0	0	0	0	0	0	0
BO04	51,557473	6,869515	08.04.19	22.04.20	Ebersbach	Rhine	near-natural	0	0	0	0	0	0	0	0
BO05	51,537199	6,857960	08.04.19	22.04.20	Rotbach	Rhine	near-natural	0	0	0	0	0	0	0	0
BO08	51,559560	6,983006	02.04.19	21.04.20	Wittringer Mühlenbach	Boye (Emscher)	restored 2010	0	0	0	0	0	0	0	0
BO12	51,542215	6,939166	29.03.19	21.04.20	Kirchschemmsbach	Boye (Emscher)	restored 2007	0	0	0	0	0	0	0	0
BO13	51,562628	6,955543	29.03.19	20.04.20	Haarbach	Boye (Emscher)	restored 2011	0	0	0	0	0	0	0	0
BO14	51,570292	6,960856	29.03.19	20.04.20	Haarbach	Boye (Emscher)	restored 2011	0	0	0	0	0	0	0	0
BO19	51,571629	6,944156	04.04.19	20.04.20	Alter Haarbach	Boye (Emscher)	near-natural	0	0	0	0	0	0	0	0
BO28	51,587987	6,943634	01.04.19	16.04.20	Brabecker Mühlenbach	Boye (Emscher)	near-natural	0	0	0	0	0	0	0	0
BO29	51,598736	6,939163	08.04.19	16.04.20	Bornemannsbach	Boye (Emscher)	near-natural	0	0	0	0	0	0	0	0
BO30	51,592411	6,932909	05.04.19	16.04.20	Wiesentalbach	Boye (Emscher)	near-natural	0	0	0	0	0	0	0	0
BE02	51,481699	6,925260	20.03.19	15.04.20	Barchembach	Emscher	near-natural	0	0	0	0	0	0	0	0
BE04	51,477657	6,903294	25.03.19	15.04.20	Heilgraben	Emscher	near-natural	0	0	0	0	0	0	0	0
BE05	51,468529	6,909343	25.03.19	15.04.20	Hexbach	Emscher	near-natural	0	0	0	0	0	0	0	0
BE14	51,413991	6,957609	28.03.19	15.04.20	Steinbach	Ruhr	near-natural	0	0	0	0	0	0	0	0
BE15	51,409337	6,952697	26.03.19	15.04.20	Ruhmbach	Ruhr	near-natural	0	0	0	0	0	0	0	0
BE16	51,406501	6,985709	26.03.19	27.04.20	Wolfsbach	Ruhr	near-natural	0	0	0	0	0	0	0	0
BE32	51,421979	6,973276	22.03.19	27.04.20	Kesselbach	Berne (Emscher)	near-natural	0	0	0	0	0	0	0	0
BE34	51,421128	6,990172	22.03.19	27.04.20	Borbecker Mühlenbach	Berne (Emscher)	near-natural	0	0	0	0	0	0	0	0
BE35	51,417001	6,986024	22.03.19	27.04.20	Borbecker Mühlenbach	Berne (Emscher)	near-natural	0	0	0	0	0	0	0	0
								**52 (59)**	**51 (51)**	**72 (83)**	**64 (65)**	**63 (75)**	**66 (68)**	**136 (140)**	**94 (96)**
