# Supplementary material for: Strong Small‐Scale Differentiation but No Cryptic Species Within the Two Isopod Species Asellus aquaticus and Proasellus coxalis in a Restored Urban River System (Emscher, Germany)
Source: Ecol Evol. 2024 Nov 18;14(11):e70575. doi: 10.1002/ece3.70575 (PMC11573423; doi:10.1002/ece3.70575)
Supplement: Supplementary file 3 — Table S3. Haplotype distribution for A. aquaticus . Given are numbers for each year and for both years together (indicated with gray background). [file ECE3-14-e70575-s011.pdf]

Tab. S3: Haplotype distribution for *A. aquaticus.* Given are numbers for each year and for both years together (indicated with grey background).

site	n	H1	H2	H3	H4	H5	H6	H7	H8	H9	H10	H11	H12	H13	H14	H15	H16	H17	H18	H19
BE29_19	10	4	1	0	0	0	2	1	2	0	0	0	0	0	0	0	0	0	0	0
BE29_20	7	1	2	0	0	0	3	0	0	0	0	0	0	0	0	0	0	1	0	0
**BE29**	17	5	3	0	0	0	5	1	2	0	0	0	0	0	0	0	0	1	0	0
BE30_19	9	5	2	0	0	0	1	0	1	0	0	0	0	0	0	0	0	0	0	0
BE30_20	13	8	1	0	0	0	4	0	0	0	0	0	0	0	0	0	0	0	0	0
**BE30**	22	13	3	0	0	0	5	0	1	0	0	0	0	0	0	0	0	0	0	0
BE31_19	3	1	0	0	0	0	1	0	1	0	0	0	0	0	0	0	0	0	0	0
BE31_20	13	10	1	0	0	0	2	0	0	0	0	0	0	0	0	0	0	0	0	0
**BE31**	16	11	1	0	0	0	3	0	1	0	0	0	0	0	0	0	0	0	0	0
BO00_19	8	4	2	0	0	0	0	0	1	0	0	0	0	0	0	1	0	0	0	0
BO00_20	15	7	6	0	0	0	0	0	1	0	1	0	0	0	0	0	0	0	0	0
**BO00**	23	11	8	0	0	0	0	0	2	0	1	0	0	0	0	1	0	0	0	0
**BO02_20**	3	2	0	0	0	0	0	0	0	0	0	0	0	0	0	0	1	0	0	0
BO07_19	4	2	0	0	0	0	0	0	0	0	0	0	2	0	0	0	0	0	0	0
BO07_20	13	11	1	0	0	0	0	0	0	0	0	0	1	0	0	0	0	0	0	0
**BO07**	17	13	1	0	0	0	0	0	0	0	0	0	3	0	0	0	0	0	0	0
**BO09_19**	1	0	0	1	0	0	0	0	0	0	0	0	0	0	0	0	0	0	0	0
**BO11_20**	15	3	10	0	0	0	0	0	0	0	0	0	0	0	0	1	0	0	0	1
BO15_19	9	5	3	0	0	0	0	0	1	0	0	0	0	0	0	0	0	0	0	0
BO15_20	14	6	5	0	0	0	0	0	3	0	0	0	0	0	0	0	0	0	0	0
**BO15**	23	11	8	0	0	0	0	0	4	0	0	0	0	0	0	0	0	0	0	0
BO16_19	2	2	0	0	0	0	0	0	0	0	0	0	0	0	0	0	0	0	0	0
BO16_20	11	6	5	0	0	0	0	0	0	0	0	0	0	0	0	0	0	0	0	0
**BO16**	13	8	5	0	0	0	0	0	0	0	0	0	0	0	0	0	0	0	0	0
BO24_19	9	0	0	0	0	0	0	0	0	2	0	1	0	6	0	0	0	0	0	0
BO24_20	16	0	0	0	2	0	0	0	0	3	0	0	0	10	0	0	0	1	0	0
**BO24**	25	0	0	0	2	0	0	0	0	5	0	1	0	16	0	0	0	1	0	0
**BO25_20**	5	1	0	0	0	0	0	0	0	0	0	0	0	0	0	0	0	3	1	0
BO27_19	8	0	0	0	3	3	0	0	0	0	0	0	2	0	0	0	0	0	0	0
BO27_20	11	0	0	0	3	2	0	0	0	0	1	0	3	0	2	0	0	0	0	0
**BO27**	19	0	0	0	6	5	0	0	0	0	1	0	5	0	2	0	0	0	0	0
Sum	199	78	39	1	8	5	13	1	10	5	2	1	8	16	2	2	1	5	1	1
