# Supplementary material for: Strong Small‐Scale Differentiation but No Cryptic Species Within the Two Isopod Species Asellus aquaticus and Proasellus coxalis in a Restored Urban River System (Emscher, Germany)
Source: Ecol Evol. 2024 Nov 18;14(11):e70575. doi: 10.1002/ece3.70575 (PMC11573423; doi:10.1002/ece3.70575)
Supplement: Supplementary file 4 — Table S4. Results for the best partitions found by ASAP using K80 as a substitution model for A. aquaticus and P. coxalis . [file ECE3-14-e70575-s002.pdf]

Tab. S4: Results for the best partitions found by ASAP using K80 as a substitution model for *A. aquaticus* and *P. coxalis*.

species	Nb of species	asap-score	P-val (rank)	W (rank)	Treshold dist.
A. aquaticus	2	1,00	2.61e-03 (1)	5.09e-05 (1)	0,06937
	12	3,50	1.56e-01 (4)	1.22e-05 (3)	0,00536
	7	3,50	1.68e-01 (5)	1.43e-05 (2)	0,00984
	6	5,00	1.22e-01 (3)	3.47e-06 (7)	0,01164
	10	5,00	1.86e-01 (6)	7.90e-06 (4)	0,00804
	4	6,00	3.71e-01 (7)	3.98e-06 (5)	0,01348
	19	6,50	1.80e-02 (2)	4.46e-07 (11)	0,00089
	3	7,50	4.23e-01 (9)	3.49e-06 (6)	0,01804
	13	8,50	3.77e-01 (8)	1.25e-06 (9)	0,00267
	5	9,00	5.07e-01 (10)	2.46e-06 (8)	0,01255
P. coxalis	3	1,50	7.16e-04 (2)	1.37e-04 (1)	0,00968
	3	2,50	1.00e-05 (1)	1.57e-05 (4)	0,03579
	4	3,50	3.83e-02 (4)	1.79e-05 (3)	0,00676
	5	3,50	5.80e-02 (5)	7.67e-05 (2)	0,00482
	6	4,00	3.44e-02 (3)	1.52e-06 (5)	0,00193
