# Supplementary material for: Strong Small‐Scale Differentiation but No Cryptic Species Within the Two Isopod Species Asellus aquaticus and Proasellus coxalis in a Restored Urban River System (Emscher, Germany)
Source: Ecol Evol. 2024 Nov 18;14(11):e70575. doi: 10.1002/ece3.70575 (PMC11573423; doi:10.1002/ece3.70575)
Supplement: Supplementary file 5 — Table S5. Different measures for genetic diversity for both species per sampling site. Diversity measures for ddRAD are as follows: allelic richness (AR) and observed heterozygosity (H O). Measures for COI are haplotype diversity (HDiv) and nucleotide diversity (NDiv). n is the number of specimens for each marker. Diversity was only calculated for sites with n > 5. [file ECE3-14-e70575-s009.pdf]

Tab. S5: Different measures for genetic diversity for both species per sampling site. Diversity measures for ddRAD are: allelic richness (AR) and observed heterozygosity (Ho). Measures for COI are haplotype diversity (HDiv) and nucleotide diversity (NDiv). n is the number of specimens for each marker. Diversity was only calculated for sites with n > 5.)

species	site	status site	ddRAD			COI		
			n	AR	Ho	n	HDiv	NDiv
A. aquaticus	BO00	restored (2020)	15	1,47	0,12	23	0,668	0,0085
	BE30	restored (2011)	18	1,46	0,13	22	0,606	0,0067
	BE29	restored (2012)	15	1,46	0,13	17	0,824	0,0234
	BO15	restored (2011)	16	1,45	0,13	23	0,648	0,0073
	BO24	near-natural	21	1,44	0,12	25	0,563	0,0212
	BE31	near-natural	13	1,43	0,12	16	0,517	0,0066
	BO25	near-natural	7	1,42	0,13	5	0,700	0,0468
	BO16	restored (1993)	10	1,42	0,12	13	0,513	0,0009
	BO11	restored (2008)	8	1,39	0,12	15	0,543	0,0205
	BO07	restored (2011)	17	1,39	0,12	17	0,404	0,0085
	BO27	near-natural	16	1,38	0,11	19	0,790	0,0184
P. coxalis	BE20	waste-water free	15	1,45	0,14	15	0,248	0,0143
	BE21	waste-water free	16	1,45	0,14	22	0,000	0,0000
	BO17	restored (1993)	19	1,46	0,14	18	0,000	0,0000
	BO23	near-natural	16	1,48	0,14	20	0,268	0,0165
	BO26	near-natural	16	1,43	0,13	16	0,125	0,0012
	BO27	near-natural	7	1,41	0,14	7	0,667	0,0238
	BO31	near-natural	15	1,4	0,13	14	0,000	0,0000
