# Supplementary material for: Strong Small‐Scale Differentiation but No Cryptic Species Within the Two Isopod Species Asellus aquaticus and Proasellus coxalis in a Restored Urban River System (Emscher, Germany)
Source: Ecol Evol. 2024 Nov 18;14(11):e70575. doi: 10.1002/ece3.70575 (PMC11573423; doi:10.1002/ece3.70575)
Supplement: Supplementary file 6 — Table S6. Summary statistics for all stacks settings for A. aquaticus and P. coxalis (Test) and for the final dataset (Final); loci limit = percentage of specimens required to have the loci, ma = minor allele frequency, H O = observed heterozygosity, H S = within‐population gene diversity, H T = overall gene diversity, and best K: K with lowest cross‐entropy (median from all repetitions) in sNMF analysis. [file ECE3-14-e70575-s003.pdf]

Tab. S6: Summary statistics for all stacks settings for *A. aquaticus* and *P. coxalis* (Test) and for the final dataset (Final); loci limit = percentage of specimens required to have the loci, ma = minor allele frequency, H_O = observed heterozygosity, H_S = within population gene diversity, H_T = overall gene diversity, best K: K with lowest cross-entropy (median from all repetitions) in sNMF analysis.

species	Test stacks settings/ Final	stacks setting	loci limit	ma	# loci	H_O	H_S	H_T	F_{ST}	F_{IS}	best K
A. aquaticus	Test	m3 M2 N4 n3	l90	ma1	2806	0.1260	0.1553	0.1689	0.0963	0.1835	5
A. aquaticus	Test	m3 M2 N4 n2	l90	ma1	2661	0.1237	0.1529	0.1664	0.0973	0.1851	5
A. aquaticus	Test	m3 M3 N5 n4	l90	ma1	2642	0.1255	0.1548	0.1683	0.0952	0.1847	4
A. aquaticus	Test	m3 M3 N5 n3	l90	ma1	2598	0.1243	0.1533	0.1669	0.0963	0.1847	5
A. aquaticus	Test	m3 M4 N6 n4	l90	ma1	2536	0.1252	0.1542	0.1678	0.0958	0.1844	5
A. aquaticus	Test	m3 M4 N6 n5	l90	ma1	2520	0.1259	0.1551	0.1689	0.0962	0.1848	4
A. aquaticus	Test	m3 M5 N7 n5	l90	ma1	2452	0.1248	0.1539	0.1677	0.0962	0.1846	5
A. aquaticus	Test	m3 M5 N7 n6	l90	ma1	2444	0.1249	0.1541	0.1677	0.0959	0.1847	4
A. aquaticus	**Final**	m3 M2 N4 n3	**l90**	**ma1**	**3302**	**0.1227**	**0.1532**	**0.1666**	**0.0961**	**0.1928**	**5**
P. coxalis	Test	m3 M3 N5 n4	l90	ma1	11764	0.1416	0.1688	0.1906	0.1442	0.1651	5
P. coxalis	Test	m3 M4 N6 n5	l90	ma1	11750	0.1404	0.1673	0.1888	0.1448	0.1637	5
P. coxalis	Test	m3 M2 N4 n3	l90	ma1	11603	0.1387	0.1652	0.1865	0.1453	0.1624	5
P. coxalis	Test	m3 M5 N7 n6	l90	ma1	11589	0.1420	0.1694	0.1913	0.1442	0.1654	5
P. coxalis	Test	m3 M5 N7 n5	l90	ma1	11424	0.1411	0.1684	0.1901	0.144	0.1662	5
P. coxalis	Test	m3 M4 N6 n4	l90	ma1	11381	0.1406	0.1676	0.1892	0.1447	0.165	5
P. coxalis	Test	m3 M3 N5 n3	l90	ma1	11038	0.1384	0.1654	0.1868	0.145	0.1664	5
P. coxalis	Test	m3 M2 N4 n2	l90	ma1	9848	0.1344	0.1613	0.1822	0.1458	0.1661	5
P. coxalis	**Final**	**m3 M3 N5 n4**	**l90**	**ma1**	**12186**	**0.1402**	**0.168**	**0.1898**	**0.1455**	**0.168**	**5**
