# Supplementary material for: Strong Small‐Scale Differentiation but No Cryptic Species Within the Two Isopod Species Asellus aquaticus and Proasellus coxalis in a Restored Urban River System (Emscher, Germany)
Source: Ecol Evol. 2024 Nov 18;14(11):e70575. doi: 10.1002/ece3.70575 (PMC11573423; doi:10.1002/ece3.70575)
Supplement: Supplementary file 7 — Table S7. Haplotype distribution for P. coxalis . Given are numbers for each year and for both years together (indicated with gray background). [file ECE3-14-e70575-s010.pdf]

Tab. S7: Haplotype distribution for *P. coxalis.* Given are numbers for each year and for both years together (indicated with grey background).

site	n	H1	H2	H3	H4	H5	H6
BE20_19	**9**	**7**	0	0	**2**	0	0
BE20_20	**6**	**6**	0	0	0	0	0
**BE20**	**15**	**13**	0	0	**2**	0	0
BE21_19	**9**	**9**	0	0	0	0	0
BE21_20	**13**	**13**	0	0	0	0	0
**BE21**	**22**	**22**	0	0	0	0	0
BO07_19	**3**	**3**	0	0	0	0	0
BO07_20	**1**	**1**	0	0	0	0	0
**BO07**	**4**	**4**	0	0	0	0	0
**BO16_20**	**1**	**1**	0	0	0	0	0
BO17_19	**5**	**5**	0	0	0	0	0
BO17_20	**13**	**13**	0	0	0	0	0
**BO17**	**18**	**18**	0	0	0	0	0
**BO20_19**	**4**	**4**	0	0	0	0	0
**BO21_20**	**2**	**1**	**1**	0	0	0	0
BO23_19	**8**	**7**	0	0	0	**1**	0
BO23_20	**12**	**10**	0	0	0	**2**	0
**BO23**	**20**	**17**	0	0	0	**3**	0
**BO25_20**	**1**	**1**	0	0	0	0	0
BO26_19	**7**	0	0	0	0	**7**	0
BO26_20	**9**	0	0	0	0	**8**	**1**
**BO26**	**16**	0	0	0	0	**15**	**1**
**BO27_20**	**7**	**2**	0	**4**	0	**1**	0
BO31_19	**7**	0	0	0	**7**	0	0
BO31_20	**7**	0	0	0	**7**	0	0
**BO31**	**14**	0	0	0	**14**	0	0
Sum	**124**	**83**	**1**	**4**	**16**	**19**	**1**
