# Supplementary material for: Strong Small‐Scale Differentiation but No Cryptic Species Within the Two Isopod Species Asellus aquaticus and Proasellus coxalis in a Restored Urban River System (Emscher, Germany)
Source: Ecol Evol. 2024 Nov 18;14(11):e70575. doi: 10.1002/ece3.70575 (PMC11573423; doi:10.1002/ece3.70575)
Supplement: Supplementary file 8 — Figure S1. FST heatmaps for pairwise comparisons between sampling sites for A. aquaticus (A, B) and P. coxalis (C, D) and COI (A, C) and ddRAD data (B, D), respectively. Above the diagonal pairwise F ST values are given and below either p values (COI data sets; values < 0.05 indicate significant differentiation) or the lower confidence interval (ddRAD data set; values > 0 indicate significant differentiation) is given. In the diagonal, F ST values for the comparison between the years 2019 and 2020 are given, with a white square, when only samples from 1 year were available. Significant F ST values are indicated in bold and all values are colored according to the level of differentiation. [file ECE3-14-e70575-s008.pdf]

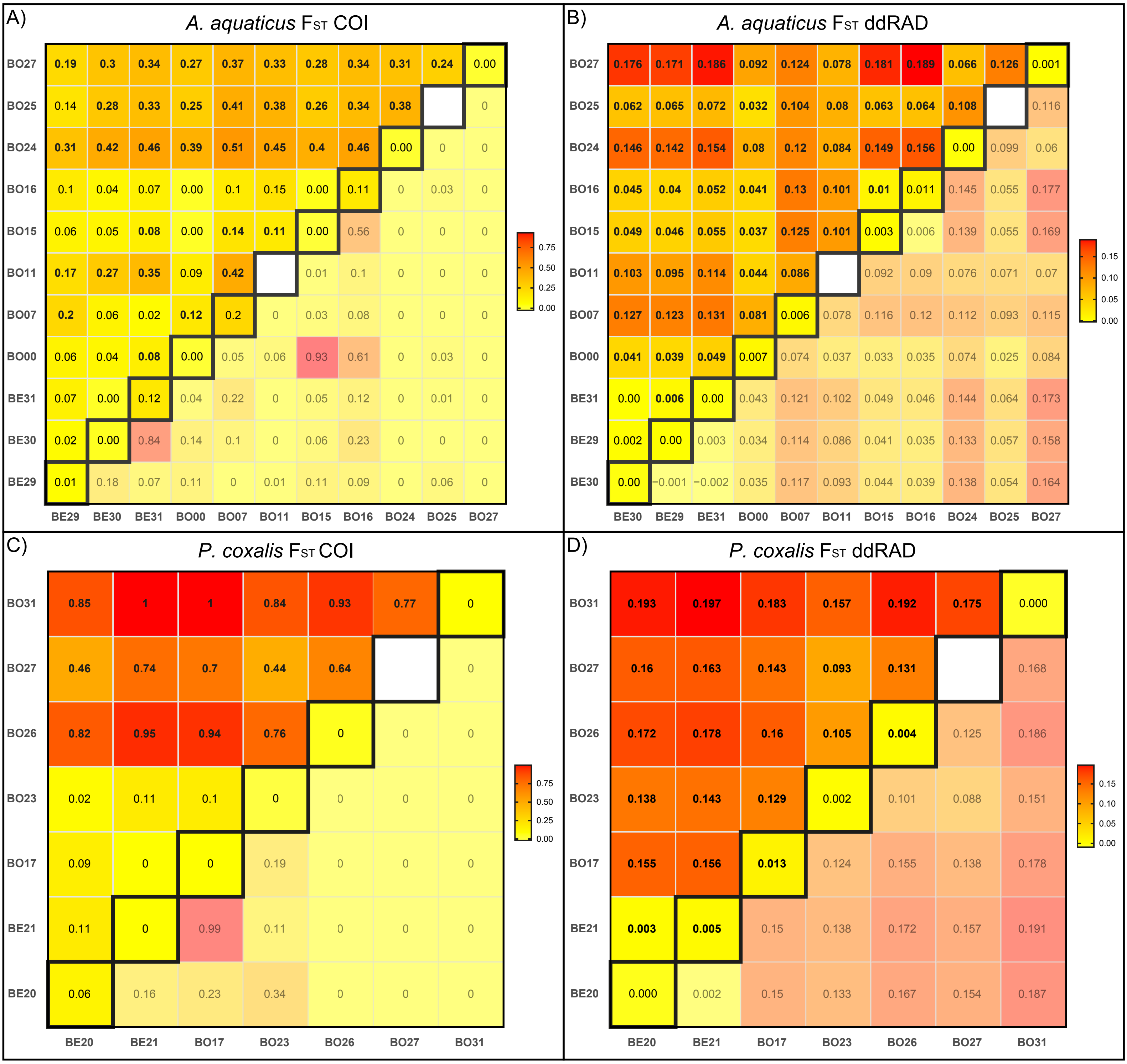

Fig. S1: F_{ST} heat maps for pairwise comparisons between sampling sites for *A. aquaticus* (A, B) and *P. coxalis* (C, D) and COI (A, C) and ddRAD data (B, D), respectively. Above the diagonal pairwise F_{ST} values are given and below either p-values (COI data sets; values < 0.05 indicate significant differentiation) or the lower confidence interval (ddRAD data set; values > 0 indicate significant differentiation) are given. In the diagonal, F_{ST} values for the comparison between the years 2019 and 2020 are given, with a white square, when only samples from one year were available. Significant F_{ST} values are indicated in bold and all values are colored according to the level of differentiation and negative values were set to 0.
