# Supplementary material for: Strong Small‐Scale Differentiation but No Cryptic Species Within the Two Isopod Species Asellus aquaticus and Proasellus coxalis in a Restored Urban River System (Emscher, Germany)
Source: Ecol Evol. 2024 Nov 18;14(11):e70575. doi: 10.1002/ece3.70575 (PMC11573423; doi:10.1002/ece3.70575)
Supplement: Supplementary file 9 — Figure S2. Correlation between pairwise genetic distances (F ST; A and B COI data, C and D ddRAD data) and waterway distances for A. aquaticus (A, C) and P. coxalis (B, D). [file ECE3-14-e70575-s006.pdf]

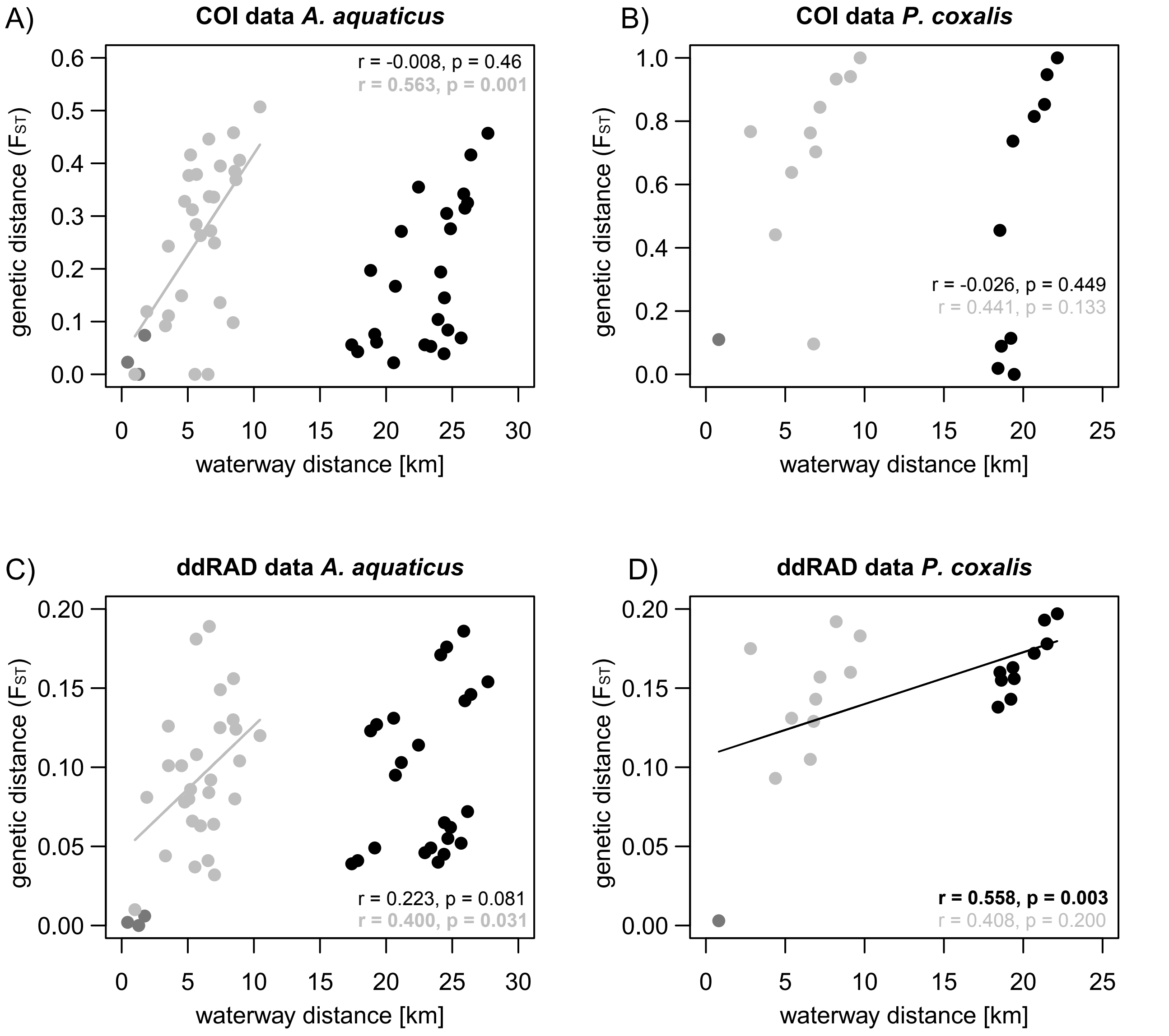

Fig. S2: Correlation between pairwise genetic distances (F_{ST}; A and B COI data, C and D ddRAD data) and waterway distances for *A. aquaticus* (A, C) and *P. coxalis* (B, D).
