# Supplementary material for: Strong Small‐Scale Differentiation but No Cryptic Species Within the Two Isopod Species Asellus aquaticus and Proasellus coxalis in a Restored Urban River System (Emscher, Germany)
Source: Ecol Evol. 2024 Nov 18;14(11):e70575. doi: 10.1002/ece3.70575 (PMC11573423; doi:10.1002/ece3.70575)
Supplement: Supplementary file 11 — Figure S4. PCA of the ddRAD data of (A) A. aquaticus and (D) P. coxalis , with the first plot showing the first and the second axes and the second plot showing the first and the third axes, respectively. (B, C) Show standard boxplots of cross‐entropy values (40 repeats) of sNMF analysis for final ddRAD datasets for A. aquaticus and P. coxalis , respectively. [file ECE3-14-e70575-s014.pdf]

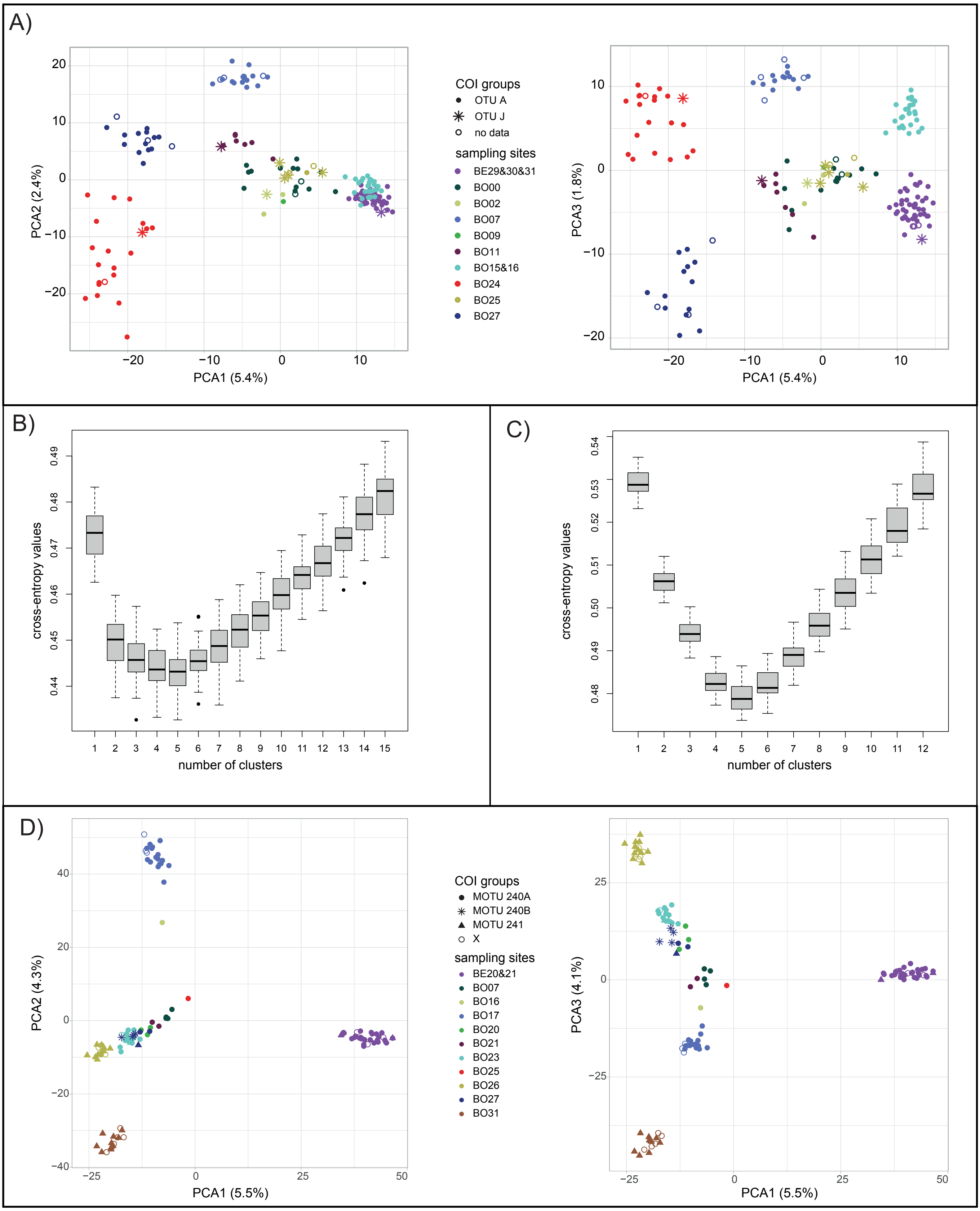

Fig. S4: PCA of the ddRAD data of A) *A. aquaticus* and D) *P. coxalis*, with the first plot showing the 1st and the 2nd axis and the second plot showing the 1st and the 3rd axis, respectively. B) and C) show standard boxplots of cross-entropy values (40 repeats) of sNMF analysis for final ddRAD datasets for *A. aquaticus* and *P. coxalis*, respectively.
