# Supplementary material for: Strong Small‐Scale Differentiation but No Cryptic Species Within the Two Isopod Species Asellus aquaticus and Proasellus coxalis in a Restored Urban River System (Emscher, Germany)
Source: Ecol Evol. 2024 Nov 18;14(11):e70575. doi: 10.1002/ece3.70575 (PMC11573423; doi:10.1002/ece3.70575)
Supplement: Supplementary file 12 — Figure S5. Relative migration networks (D) for (A) A. aquaticus and (B) P. coxalis . Only populations with > 5 individuals were included in the analysis. [file ECE3-14-e70575-s001.pdf]

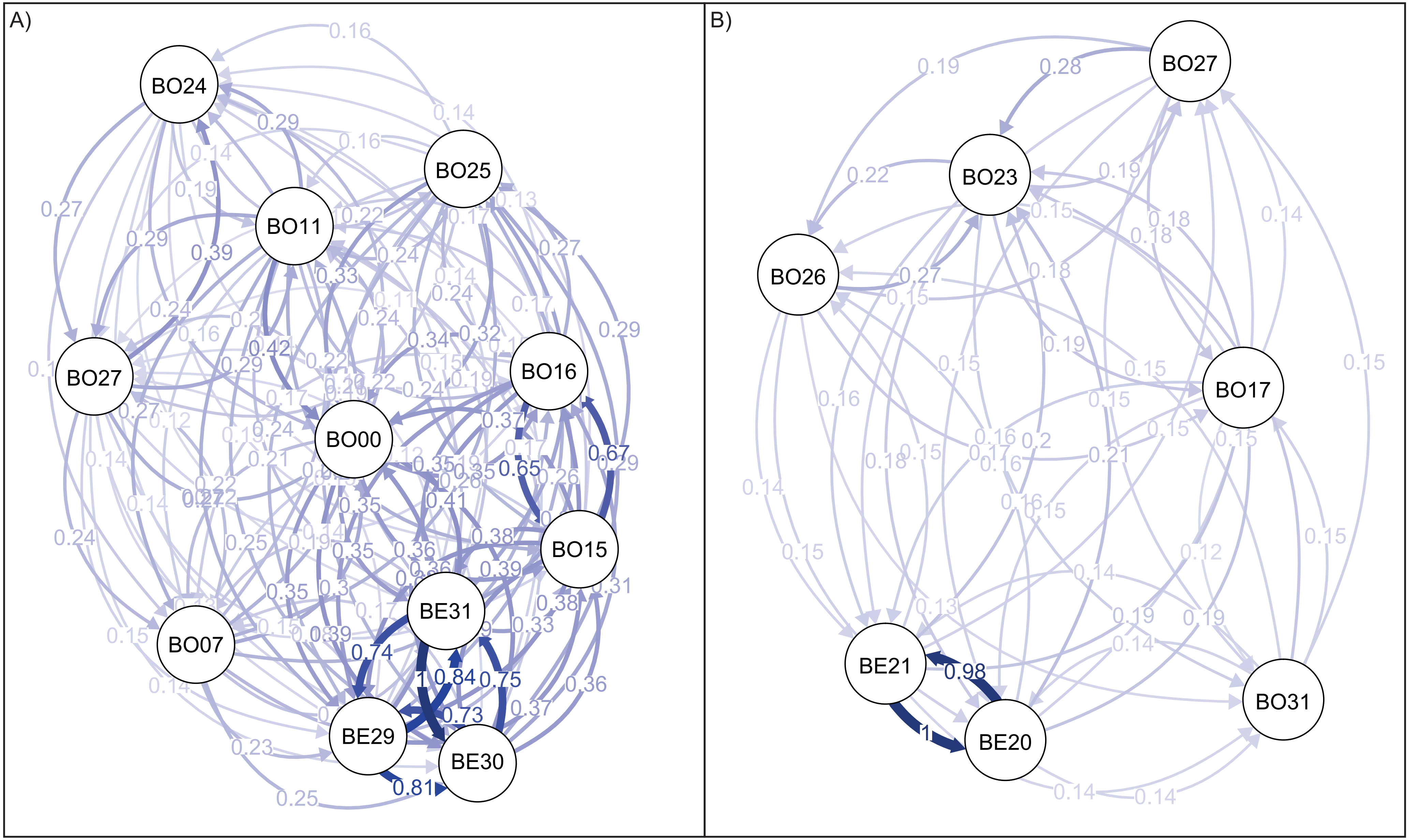


**Fig. S5**: Relative migration networks (D) for A) *A. aquaticus* and B) *P. coxalis*. Only populations with >5 individuals were included in the analysis.
