# Supplementary material for: Strong Small‐Scale Differentiation but No Cryptic Species Within the Two Isopod Species Asellus aquaticus and Proasellus coxalis in a Restored Urban River System (Emscher, Germany)
Source: Ecol Evol. 2024 Nov 18;14(11):e70575. doi: 10.1002/ece3.70575 (PMC11573423; doi:10.1002/ece3.70575)
Supplement: Supplementary file 13 — Figure S6. Neighbor Net of P. coxalis for the ddRAD data set. Branches are colored according to sampling sites. [file ECE3-14-e70575-s013.pdf]

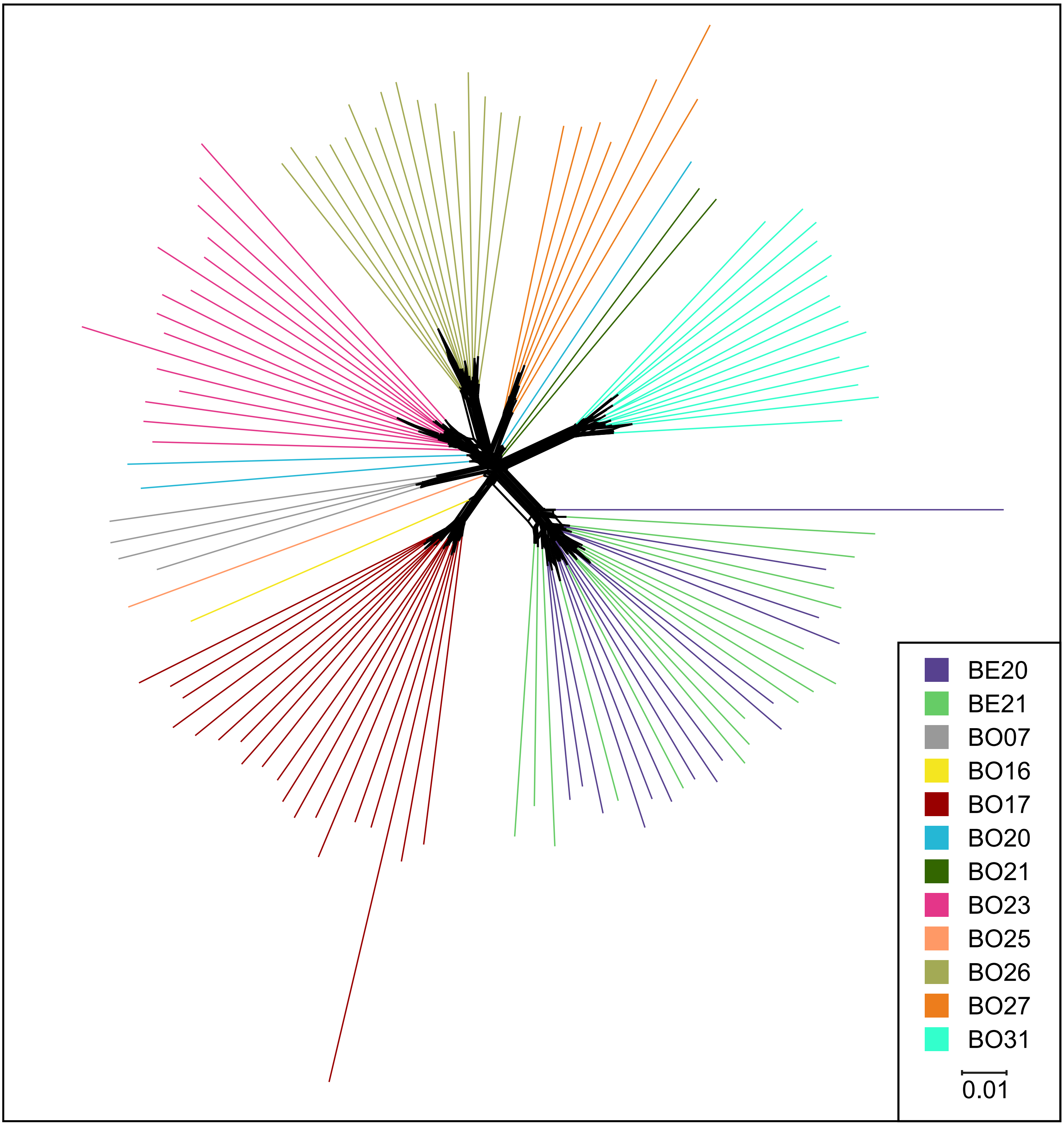

Fig. S6: Neighbor net of *P. coxalis* for the ddRAD data set. Branches are colored according to sampling sites.
